# β-blockers and risk of neuropsychiatric adverse events: An active-comparator restricted disproportionality on the FAERS

**DOI:** 10.1177/02698811251349190

**Published:** 2025-07-24

**Authors:** Lujain Ez Eddin, Mohammad Ali Omrani, Niaz Chalabianloo, Flory T Muanda

**Affiliations:** 1 ICES Western, London, ON, Canada; 2 Department of Physiology and Pharmacology, Western University, London, ON, Canada; 3 Department of Computer Science, Western University, London, ON, Canada; 4 Department of Epidemiology and Biostatistics, Western University, London, ON, Canada; 5 Lawson Health Research Institute, London Health Sciences Center, London, ON, Canada

**Keywords:** β-blockers, disproportionality analysis, neuropsychiatric disorders

## Abstract

**Background:**

β-blockers (β-adrenoceptor antagonists), commonly used for cardiovascular conditions, may be linked to neuropsychiatric adverse events (AEs). However, many prevalent ones, including delirium and hallucinations, remain insufficiently studied.

**Aims:**

To compare the neuropsychiatric risks of β-blockers with other antihypertensive drugs using data from the FDA Adverse Event Reporting System (FAERS) and differences between lipophilic and hydrophilic β-blockers.

**Method:**

An active-comparator restricted disproportionality analysis was conducted using data from the FAERS (2004Q1–2023Q4). Neuropsychiatric AEs were analyzed using Preferred Terms and the System Organ Classes from the Medical Dictionary for Regulatory Activities for β-blockers compared to lisinopril and losartan. Adjusted Reporting Odds Ratios (aRORs) were calculated using logistic regression to account for potential confounders.

**Results:**

β-blockers were linked to a significantly higher risk of nervous and psychiatric disorders, compared to lisinopril and losartan. Among the nine types of neuropsychiatric events studied, six—dizziness, nightmares, insomnia, hallucinations, somnolence, and disorientation—showed higher aRORs with β-blockers. Propranolol, a lipophilic β-blocker, exhibited the highest aRORs for psychiatric disorders and six types of neuropsychiatric events, including nightmares, delirium, hallucinations, disorientation, altered mental status, and somnolence, compared to lisinopril and losartan. Compared to atenolol, propranolol remained significantly associated with delirium, hallucinations, and disorientation.

**Conclusion:**

β-blockers, especially propranolol, may be associated with a higher risk of neuropsychiatric AEs compared to lisinopril and losartan. These findings highlight the importance of considering the specific β-blocker prescribed, particularly in patients at risk for central nervous system side effects. Further population-based studies are warranted to confirm these results.

## Introduction

β-blockers (β-adrenoceptor antagonists) are essential in treating a range of cardiovascular diseases, particularly coronary heart disease and hypertension (Ziff et al., 2020). By inhibiting β-adrenergic receptors, these drugs reduce heart rate, myocardial contractility, and oxygen demand. Given their significant therapeutic effects, β-blockers are widely prescribed for long-term management. However, their use is associated with various adverse events (AEs) that may impact treatment adherence and overall patient well-being (Poirier and Tobe, 2014; Zeitouni et al., 2019). While cardiovascular adverse effects such as bradycardia and hypotension are well-known, there is increasing concern about the potential neuropsychiatric AEs associated with β-blockers (Lama, 2002). Symptoms such as dizziness, insomnia, and nightmares, along with more severe conditions such as depression and cognitive impairment, have been reported. These neuropsychiatric effects can have a profound impact on patients, particularly when these medications are used for extended periods (McAinsh and Cruickshank, 1990).

The likelihood of β-blockers causing neuropsychiatric effects may be influenced by their pharmacokinetic properties. Lipophilic β-blockers are more likely to cross the blood–brain barrier and interact with central nervous system (CNS) receptors (Cojocariu et al., 2021), potentially increasing the risk of neuropsychiatric adverse effects. Among the β-blockers included in our study, propranolol has the highest lipophilicity. Metoprolol and carvedilol are considered to have moderate lipophilicity and may also penetrate the CNS to a meaningful extent. In contrast, atenolol is hydrophilic and has limited CNS penetration (Cojocariu et al., 2021). These pharmacokinetic differences could explain varying neuropsychiatric side effect profiles across these agents.

While a previous study using disproportionality analysis found that lipophilic β-blockers were more frequently associated with reports of nightmares, suggesting that CNS penetration could contribute to these AEs, many other common neuropsychiatric AEs, such as cognitive impairment, insomnia, dizziness, delirium, disorientation, somnolence, and hallucinations, have not been thoroughly examined (Garcia et al., 2021). We recently conducted a systematic review and meta-analysis, which suggested a potential link between β-blocker use, particularly lipophilic β-blockers, and an increased risk of common neuropsychiatric AEs listed above (Eddin et al., 2024). However, these findings should be interpreted with caution due to the inclusion of outdated studies and a high risk of bias in most of the studies, which limits the strength of the conclusions and makes these results inconclusive.

To fill this gap, we conducted a disproportionality analysis using the FDA Adverse Event Reporting System (FAERS) database to examine whether β-blockers are associated with common neuropsychiatric AEs.

This study aims to investigate two central hypotheses: whether β-blockers are associated with a higher risk of neuropsychiatric AEs compared to other antihypertensive medications such as lisinopril (an angiotensin-converting enzyme (ACE) inhibitors) and losartan (an angiotensin II receptor blockers (ARB)), and whether lipophilic β-blockers carry a higher risk of neuropsychiatric events compared to hydrophilic β-blockers.

To address these hypotheses, we performed an active-comparator restricted disproportionality analysis to compare β-blockers (drug of interest) to an active-comparator group (other antihypertensive agents such as lisinopril and losartan). By restricting the analysis to active comparators, the study focused on drugs used for similar conditions and in comparable patient populations. This approach minimizes confounding factors related to indications or comorbidities, thereby reducing the likelihood of false-positive associations (Alkabbani and Gamble, 2023). In this study, the analysis compared the odds of neuropsychiatric AEs reported for β-blockers against those reported for lisinopril and losartan. In addition, the study evaluated whether the reports of these events differ between lipophilic and hydrophilic β-blockers.

## Methods

### Study design

An active-comparator restricted disproportionality analysis was conducted using individual case safety reports (ICSRs) from the FAERS database to evaluate neuropsychiatric AEs associated with β-blockers users, compared to patients taking losartan and lisinopril. The β-blockers assessed included atenolol, propranolol, carvedilol, and metoprolol, which are among the most frequently used in clinical practice (Gu et al., 2010; Mahfoud et al., 2024). Other beta-blockers in the FAERS database, such as acebutolol, bisoprolol, nebivolol, and sotalol, were not included in the analysis, as they are less commonly prescribed and may not accurately represent the typical usage patterns seen in clinical settings. The focus on the most commonly used β-blockers ensures that the results are reflective of general clinical practice and enhances the external validity of the findings.

All included ICSRs were analyzed regardless of the reported indication for use of the study medications. Non-cardiovascular uses of β-blockers—such as for migraine, anxiety, and hemangiomas—were not excluded from the primary analysis, due to limitations in the completeness and consistency of indication data in FAERS.

In addition, an analysis was performed between propranolol and atenolol to examine differences in neuropsychiatric reports between lipophilic and hydrophilic β-blockers.

In this study, our primary analysis was restricted to β-blockers (atenolol, propranolol, carvedilol, metoprolol), losartan (an ARB), and lisinopril (an ACE inhibitor) to reduce concerns about confounding by indications, given their similar clinical indications, particularly for managing hypertension and related cardiovascular conditions. We specifically chose lisinopril and losartan as active comparators because of their widespread use, their differing pharmacological mechanisms (RAAS inhibition), and the availability of robust data in the FAERS database (Carey et al., 2018; Strauss et al., 2023). The inclusion of these agents allowed for a meaningful comparison of neuropsychiatric AEs across different drug classes with similar therapeutic applications. While other antihypertensive medications, such as calcium channel blockers (CCBs) or diuretics, could have been considered, we did not select them for several reasons. CCBs can be linked to some neuropsychiatric effects, and diuretics are often associated with electrolyte imbalances, which could confound the assessment of neuropsychiatric outcomes (Colle et al., 2007; Sardar and Eilbert, 2015).

To avoid false-positive signals, we included only cases where β-blockers, losartan, or lisinopril were identified as the primary suspect drug, ensuring the focus was on the drug most likely causing the AE based on the clinical judgment of the reporters. In addition, we adjusted for potential confounding factors, including sex, age, weight, and clinical indications such as hypertension, heart failure, and atrial fibrillation. We followed the Reporting of a Disproportionality Analysis for Drug Safety Signal Detection Using Individual Case Safety Reports in PharmacoVigilance (Appendix 3) in the Supplemental Material (Fusaroli et al., 2024).

### Data description, access, and preprocessing

This study utilized data from the FAERS, accessed through the OpenFDA (n.d.) platform. The study period encompassed AEs reported from January 1, 2004 to December 31, 2023, representing all the complete years available in the database at the time of data extraction.

The OpenFDA AEs database compiles post-marketing safety surveillance data submitted to the FDA. This data originates from FAERS, which adheres to the International Conference on Harmonization E2B guidelines for reporting safety data about post-marketing medication safety. It includes AE reports from healthcare professionals, consumers, and manufacturers. AEs are codified through the Medical Dictionary for Regulatory Activities (MedDRA, version 24.0) terminology at the preferred terms (PTs) level.

OpenFDA was preferred over raw FAERS data due to its partially preprocessed nature, which offers several advantages, including the elimination of duplicate records and provision of a more structured dataset compared to raw FAERS data. This preprocessing facilitates more efficient data handling and initial analysis, though further refinement of certain data elements, such as medication names, was still necessary for this study’s specific requirements. OpenFDA provides the data both in downloadable quarterly formats and through an Application Programming Interface, facilitating efficient querying and retrieval of large datasets (Kass-Hout et al., 2016).

The data from OpenFDA are made publicly available under the Creative Commons CC0 1.0 Universal dedication. OpenFDA ensures that the provided data contains no sensitive or personally identifiable information, precluding the need for ethics board approval. Data extraction was performed using a custom-developed script to download all available zipped JSON files from the OpenFDA platform. Despite the structured format provided by OpenFDA, extensive data cleaning was necessary due to inconsistencies in drug names, particularly in the “medicinal product” field. Multiple variations in drug names were identified during a manual review, increasing usable information. We used regular expressions to normalize these names during preprocessing and matched them against databases such as RxNorm and Observational Health Data Sciences and Informatics (Hripcsak et al., 2015). We included generic names, Anatomical Therapeutic Chemical classification system codes, and RxNorm Concept Unique Identifiers to further enhance the collection (RxNorm, n.d.). It has been simplified to identify primary suspect drugs, secondary suspect drugs, interacting drugs, and concurrent drugs by mapping the drug characterization variables in OpenFDA to the role code field in FAERS. By comparing the safety report ID numbers associated with each report, duplicate cases were found and eliminated, guaranteeing that the final dataset was clear and concentrated on the main adverse drug events (ADE) suspects.

### Variables definition

The study population included all ICSR from FAERS between January 1, 2004 and December 31, 2023, containing β-blockers (atenolol, propranolol, carvedilol, and metoprolol), losartan (an ARB), or lisinopril (an ACE inhibitor) reports during the study period. The primary analysis was restricted to these medications to reduce concerns about confounding by indications, given their similar clinical uses, particularly for managing hypertension and related cardiovascular conditions, and to avoid false-positive signals. Only cases where β-blockers, losartan, or lisinopril were identified as the primary suspect drug were included in the analysis. We wanted to include only reports in which the reporter had already suspected the drug of interest as the primary cause of the AE, ensuring a focus on the most likely drug-related ADEs based on the clinical judgment of the reporter. Key variables assessed included demographic factors (e.g., age, sex, and weight) and clinical indications (e.g., hypertension, heart failure, and atrial fibrillation), which are known risk factors for ADEs (Alomar, 2014). Adjustments for these variables were performed to account for potential confounding and to ensure the robustness of the findings.

Time-to-onset of ADEs was calculated as the time between the first administration of the drug of interest and the event date, when the information was available. Grouping of drugs was based on their active ingredients (e.g., atenolol, propranolol, carvedilol, metoprolol, losartan, or lisinopril) identified after the cleaning and the standardization of the medicinal product field to minimize misclassification.

We searched for specific neuropsychiatric ADEs using the PTs from the MedDRA, including dizziness, nightmares, insomnia, confusional state, altered mental status, hallucinations, disorientation, delirium, and somnolence. In addition, we performed a broader analysis using the System Organ Classes for “Nervous System Disorders” and “Psychiatric Disorders” to capture any related neuropsychiatric events comprehensively.

### Statistical analysis

The characteristics of ICSR for β-blockers (as a group and individually for atenolol, propranolol, metoprolol, and carvedilol), as well as lisinopril and losartan, were described using the median and interquartile range (IQR) for continuous variables, as these measures are more appropriate for skewed or non-normally distributed data. Numbers and proportions were used for categorical variables. The time-to-onset of the event(s) of interest was also described using the median and IQR. Disproportionality analysis in this study was conducted using the Reporting Odds Ratio (ROR) as the primary disproportionality measure. The unadjusted ROR was calculated as the odds of reporting specific neuropsychiatric ADEs with β-blockers relative to the odds of reporting the same events with lisinopril and losartan separately. A significant signal was indicated when the lower bound of the 95% confidence interval (CI) for the ROR exceeded 1.0 and the number of cases was three or more (Rothman et al., 2004; Van Puijenbroek et al., 2002). An aROR greater than 1, with CIs that do not cross 0, generally indicates statistical significance (*p* < 0.05), suggesting an increased likelihood of the drug being associated with the AE in question. Due to the spontaneous nature of reporting in FAERS and the absence of data on the total number of exposed individuals, it is not possible to estimate the incidence of AEs. Therefore, disproportionality analysis using ROR is the standard approach in pharmacovigilance research to detect potential safety signals. The adjusted ROR (aROR), which accounts for confounding variables, was considered the primary measure for assessing the relationship between drug use and ADEs.

Adjusted RORs were calculated using logistic regression to account for potential confounding factors, including sex, age, weight, and conditions such as hypertension, cardiac failure, and atrial fibrillation and were considered the primary measure for assessing the relationship between drug use and ADEs.

To further address concerns about confounding by indication, we conducted a post hoc sensitivity analysis in which we additionally adjusted for non-cardiovascular indications for beta-blocker use, such as migraine, anxiety, and hemangiomas, alongside cardiovascular indications, and demographic variables. When available, these non-cardiovascular indications were extracted from the “indication for use” field in FAERS reports.

Forest plots were generated for a visual comparison of β-blockers against active comparators. All statistical analyses were performed using SAS (Version 8.3, SAS Studio, Cary, NC, USA) and R (Version 4.3.3).

## Results

### Clinical characteristics in FAERS

After removing duplicates, we identified 90,146 reports in FAERS, where the study drugs—atenolol, propranolol, metoprolol, carvedilol, lisinopril, and losartan—were already listed as the primary suspect by the reporter. A total of 8772 reports were associated with atenolol, 9511 reports with propranolol, 19,786 reports with metoprolol, 10,671 reports with carvedilol, 27,111 reports with lisinopril, and 14,295 reports with losartan. The median age was 64 years (IQR: 49–75) for β-blockers, 64 years (IQR: 57–73) for lisinopril, and 67 years (IQR: 58–76) for losartan. Women were more frequently represented in these ICSRs, with most reports originating from the United States. Additional details are provided in Table 1.

**Table 1.** Clinical characteristics of reports associated with β-blockers: atenolol, propranolol, metoprolol, carvedilol, lisinopril, and losartan from the FAERS database from 2004Q1 to 2023Q3.

Characteristics	β-blockers (*N*, %)	Atenolol (*N*, %)	Propranolol (*N*, %)	Metoprolol (*N*, %)	Carvedilol (*N*, %)	Lisinopril (*N*, %)	Losartan (*N*, %)
Age (year)							
Median age (IQR)	64 (49–75)	66 (55–77)	37 (17–56)	66 (53–77)	68 (57–78)	64 (54–73)	67 (58–76)
<22	2390 (4.9)	225 (2.56)	1539 (16.18)	499 (2.552)	127 (1.19)	304 (1.12)	160 (1.12)
22–52	7058 (14.48)	1203 (13.71)	2127 (22.36)	2695 (13.62)	1033 (9.63)	3698 (13.64)	1253 (8.77)
52–64	6143 (12.6)	1452 (16.55)	659 (6.93)	2711 (13.7)	1321 (12.38)	5270 (19.44)	1954 (13.67)
64–75	7537 (15.46)	1681 (19.16)	515 (5.41)	3366 (17.01)	1975 (18.51)	5590 (20.62)	2747 (19.22)
75–87	6415 (13.16)	1418 (16.17)	274 (2.88)	2968 (15)	1755 (16.45)	3137 (11.57)	2135 (14.94)
>87	2093 (4.29)	647 (7.38)	64 (0.67)	999 (5.05)	383 (3.59)	876 (3.23)	459 (3.21)
Unknown	17,104 (35.09)	2146 (24.46)	4333 (45.56)	6548 (33.09)	4077 (38.21)	8236 (30.38)	5587 (39.08)
Sex							
Male	18,529 (38.02)	3423 (39.02)	2449 (25.75)	7839 (39.62)	4818 (45.15)	13,634 (40.29)	5181 (36.24)
Female	24,538 (50.34)	4762 (54.29)	5178 (54.44)	9930 (50.19)	4668 (43.74)	11,040 (4.72)	7579 (53.02)
Unknown	5673 (11.64)	587 (6.67)	1884 (19.81)	2017 (10.19)	1185 (11.1)	2437 (8.99)	1535 (10.74)
Weight (kg)							
Median weight (IQR)	72 (54–88)	75 (63–88.9)	9.5 (6.4–60.3)	77 (63.6–90.9)	78.5 (65–93.9)	85.2 (72–100.7)	78 (65–91)
<63	4690 (9.62)	636 (7.25)	2359 (24.8)	1407 (7.11)	288 (2.7)	1365 (5.03)	951 (6.65)
63–79	3432 (7.04)	884 (10.08)	319 (3.35)	1810 (9.15)	419 (3.93)	2891 (10.66)	1440 (10.07)
79–82	648 (1.33)	150 (1.71)	41 (0.43)	375 (1.90)	82 (0.77)	651 (2.40)	258 (1.8)
82–94	2050 (4.21)	475 (5.41)	174 (1.83)	1159 (5.86)	242 (2.27)	2388 (8.81)	929 (6.5)
>97	2343 (4.81)	476 (5.43)	190 (2)	1334 (6.74)	343 (3.21)	3905 (14.4)	1025 (7.71)
Unknown	35,577 (72.99)	6151 (70.12)	6428 (67.58)	13,701 (69.25)	9297 (87.12)	15,911 (58.69)	9692 (67.8)
Indication of use							
Hypertension	9258 (18.99)	2803 (31.95)	408 (4.29)	3743 (18.92)	2304 (21.59)	11,977 (44.18)	6272 (43.88)
Atrial fibrillation	1190 (2.44)	222 (2.53)	59 (0.62)	753 (3.81)	156 (1.46)	20 (0.07)	12 (0.08)
Cardiac failure	1638 (3.36)	126 (1.44)	50 (0.53)	604 (3.05)	858 (8.04)	411 (1.52)	249 (1.74)
Reported person							
Physician	10,062 (20.64)	2206 (25.15)	2179 (22.91)	4287 (21.7)	1390 (13.02)	4295 (11.61)	2955 (20.67)
Pharmacist	5047 (10.35)	1178 (13.43)	1228 (12.91)	1966 (9.9)	675 (6.32)	7585 (20.49)	1398 (9.78)
Other health professionals	12,863 (26.39)	2120 (24.17)	3879 (40.78)	5058 (25.6)	1806 (16.93)	4671 (12.62)	3034 (21.23)
Lawyer	21 (0.04)	4 (0.05)	3 (0.03)	9 (0.05)	5 (0.05)	173 (0.47)	43 (0.3)
Consumer of non-health professionals	17,672 (36.26)	2557 (29.15)	1433 (15.07)	7080 (35.8)	6602 (61.89)	8473 (22.89)	6244 (43.68)
Unknown	3075 (6.31)	707 (8.06)	789 (8.3)	1386 (7)	193 (1.81)	1914 (32.19)	621 (4.34)
Reporting country							
United States	29,582 (62.23)	3751 (44.68)	5409 (58.15)	12,399 (63.49)	8023 (77.81)	21.665 (82.07)	7281 (52.3)
France	1800 (3.79)	1048 (12.48)	547 (5.88)	145 (0.74)	60 (0.58)	263 (0.99)	401 (2.88)
Rest of world	15,564 (32.74)	3546 (42.24)	3226 (34.67)	6722 (34.43)	1868 (18.12)	4415 (16.93)	5871 (42.18)
Unknown	588 (1.24)	50 (0.6)	120 (1.3)	260 (1.33)	360 (3.49)	768 (2.92)	371 (2.66)
Serious outcome							
Death	3825 (7.85)	655 (7.47)	970 (11.88)	1684 (12.22)	516 (5.07)	870 (3.02)	387 (2.37)
Life-threatening	1621 (3.33)	388 (4.42)	340 (4.16)	617 (4.48)	276 (2.71)	2074 (7.2)	467 (2.86)
Hospitalization	8802 (18.06)	2199 (25.07)	1170 (14.33)	3796 (27.53)	1637 (16.09)	6154 (21.4)	2320 (14.24)
Other	34,492 (70.76)	5530 (63.04)	6031 (73.63)	8689 (63.77)	8242 (81.13)	18,813 (65.38)	11,021 (67.53)

### Active-comparator restricted disproportionality analysis in FAERS

#### Nervous system disorder

We assessed the association between β-blockers (propranolol, atenolol, metoprolol, and carvedilol) and nervous system disorders compared to lisinopril and losartan. β-blockers showed higher RORs for nervous system disorders compared to lisinopril and losartan. Overall, β-blockers were associated with a higher risk of nervous system disorders relative to both comparators, with the strongest associations observed for atenolol and carvedilol. The median onset of nervous system disorders for β-blockers was 40 days (IQR: 4–316 days). In head-to-head analyses among β-blockers, propranolol exhibited a lower risk of nervous system disorders compared to atenolol (aROR: 0.87, 95% CI: 0.80–0.94). A summary of aRORs with 95% confidence intervals is shown in Tables 2 and 3. Forest plots are presented in the Supplemental Materials (Appendix Figures 1 and 2).

**Table 2.** Comparison of the risk of neuropsychiatric adverse events between all β-blockers and lisinopril (reference) across various adverse events^a^.

Adverse event	β-blockers, aROR (95% CI)	Propranolol, aROR (95% CI)	Atenolol, aROR (95% CI)	Carvedilol, aROR (95% CI)	Metoprolol, aROR (95% CI)
*Nervous system disorders*	2.08 (2.00–2.16)	1.83 (1.71–1.96)	2.21 (2.09–2.33)	2.16 (2.04–2.28)	1.95 (1.86–2.04)
*Psychiatric disorders*	1.68 (1.61–1.75)	1.85 (1.72–1.98)	1.69 (1.59–1.80)	1.42 (1.33–1.51)	1.67 (1.58–1.75)
*Dizziness*	1.90 (1.78–2.05)	0.76 (0.65–0.88)	1.39 (1.24–1.55)	2.90 (2.65–3.19)	1.93 (1.77–3.09)
*Nightmares*	5.67 (3.81–8.45)	5.50 (3.32–9.12)	5.41 (3.37–8.69)	4.50 (2.72–7.43)	5.27 (3.43–8.10)
*Delirium*	1.32 (0.87–2.00)	3.25 (1.62–6.52)	0.44 (0.18–1.07)	0.91 (0.47–1.76)	2.07 (1.30–3.27)
*Insomnia*	1.84 (1.61–2.10)	1.75 (1.41–2.17)	1.73 (1.44–2.09)	1.89 (1.56–2.30)	1.72 (1.47–2.01)
*Hallucination*	1.59 (1.28–1.97)	2.46 (1.74–3.50)	0.93 (0.66–1.31)	0.74 (0.51–1.08)	1.91 (1.49–2.44)
*Somnolence*	2.50 (2.10–2.99)	2.80 (2.16–3.63)	2.00 (1.57–2.56)	3.07 (2.46–3.84)	2.07 (1.69–2.54)
*Disorientation*	2.02 (1.45–2.88)	2.54 (1.49–4.33)	1.51 (0.93–2.46)	1.55 (0.96–2.51)	2.07 (1.42–3.01)
*Confusion*	1.12 (0.96–1.32)	1.03 (0.76–1.41)	1.11 (0.88–1.40)	0.76 (0.58–0.99)	1.25 (1.04–1.50)
*Altered mental status*	22.79 (5.59–92.88)	31.31 (7.20–136.09)	19.22 (4.38–84.22)	14.26 (3.17–64.21)	26.83 (6.36–113.31)

a Adjusted for sex, age, weight, and conditions such as hypertension, cardiac failure, and atrial fibrillation.

**Table 3.** Comparison of the risk of neuropsychiatric adverse events between all β-blockers and losartan (reference) across various adverse events^a^.

Adverse event	β-blockers, aROR (95% CI)	Propranolol, aROR (95% CI)	Atenolol, aROR (95% CI)	Carvedilol, aROR (95% CI)	Metoprolol, aROR (95% CI)
*Nervous system disorders*	1.29 (1.23–1.34)	1.22 (1.13–1.31)	1.42 (1.34–1.51)	1.36 (1.28–1.44)	1.24 (1.18–1.31)
*Psychiatric disorders*	1.53 (1.45–1.62)	1.90 (1.75–2.07)	1.61 (1.50–1.73)	1.40 (1.31–1.51)	1.58 (1.49–1.68)
*Dizziness*	1.20 (1.11–1.29)	0.50 (0.42–0.59)	0.92 (0.82–1.03)	1.83 (1.66–2.01)	1.22 (1.12–1.33)
*Nightmares*	2.53 (1.76–3.62)	3.47 (2.13–5.65)	2.55 (1.64–3.96)	2.01 (1.25–3.22)	2.55 (1.72–3.79)
*Delirium*	0.94 (0.59–1.52)	2.41 (1.18–4.89)	0.34 (0.14–0.86)	0.81 (0.40–1.64)	1.41 (0.84–2.37)
*Insomnia*	1.44 (1.24–1.67)	1.71 (1.35–2.18)	1.44 (1.18–1.76)	1.43 (1.16–1.76)	1.38 (1.16–1.64)
*Hallucination*	1.78 (1.34–3.37)	2.65 (1.78–3.47)	1.36 (0.92–2.01)	0.76 (0.50–1.15)	2.17 (1.59–2.95)
*Somnolence*	1.73 (1.43–2.10)	2.40 (1.80–3.19)	1.41 (1.09–1.82)	2.19 (1.73–2.77)	1.50 (1.21–1.87)
*Disorientation*	1.85 (1.24–2.76)	3.87 (2.01–7.45)	1.46 (0.85–2.51)	2.02 (1.17–3.48)	2.06 (1.32–3.19)
*Confusion*	0.81 (0.68–0.96)	0.85 (0.61–1.19)	0.85 (0.67–1.09)	2.02 (1.17–3.48)	0.91 (0.75–1.11)
*Altered mental status*	1.49 (0.88–2.52)	2.58 (1.28–5.20)	1.16 (0.58–2.31)	2.02 (1.17–3.48)	1.52 (0.83–2.79)

a Adjusted for sex, age, weight, and conditions such as hypertension, cardiac failure, and atrial fibrillation.

#### Psychiatric disorder

We assessed the association between β-blockers (propranolol, atenolol, metoprolol, and carvedilol) and psychiatric disorders compared to lisinopril and losartan. β-blockers showed higher aRORs for psychiatric disorders compared to both comparators, with stronger associations observed against lisinopril than losartan. Propranolol had the highest aROR among the β-blockers, compared to lisinopril and losartan.

The median onset of psychiatric disorders for β-blockers was 24 days (IQR: 1–191 days). In head-to-head analyses among β-blockers, propranolol exhibited a nonsignificant difference in reports compared to atenolol (aROR: 0.98, 95% CI: 0.90–1.06). A summary of aRORs with 95% confidence intervals is shown in Tables 2 and 3. Forest plots are presented in the Supplemental Materials (Appendix Figures 3 and 4).

#### Dizziness

We evaluated the association between β-blockers (atenolol, propranolol, carvedilol, and metoprolol) and dizziness compared to lisinopril and losartan. β-blockers had higher RORs for dizziness relative to both comparators. Carvedilol showed the highest ROR among the β-blockers, followed by metoprolol and atenolol, while propranolol exhibited the lowest association.

The median onset of dizziness for β-blockers was 36 days (IQR: 7–279 days). In head-to-head analyses among β-blockers, propranolol showed a significantly lower association with dizziness compared to atenolol (adjusted OR: 0.73, 95% CI: 0.61–0.87). A summary of aRORs with 95% confidence intervals is shown in Tables 2 and 3. Forest plots are presented in the Supplemental Materials (Appendix Figures 5 and 6).

#### Nightmares

We assessed the risk of nightmares associated with β-blockers (propranolol, atenolol, metoprolol, and carvedilol) compared to lisinopril and losartan. β-blockers showed higher RORs for nightmares relative to both comparators. Among individual β-blockers, propranolol exhibited the highest ROR in both comparisons, followed by atenolol and metoprolol, with carvedilol showing the lowest association.

The median onset of nightmares for β-blockers was 68 days (IQR: 14–184 days). In head-to-head analyses among β-blockers, propranolol showed a nonsignificant higher risk of nightmares compared to atenolol (aROR: 1.40, 95% CI: 0.94–2.10). A summary of aRORs with 95% confidence intervals is shown in Tables 2 and 3. Forest plots are presented in the Supplemental Materials (Appendix Figures 7 and 8).

#### Delirium

We evaluated the risk of delirium associated with β-blockers (propranolol, atenolol, metoprolol, and carvedilol) compared to lisinopril and losartan. β-blockers showed no significant association with delirium compared to either lisinopril or losartan.

Among individual β-blockers, propranolol demonstrated the highest association with delirium in both comparisons. Metoprolol showed a significant association compared to lisinopril, while atenolol and carvedilol showed no significant associations in either comparison. The median onset of delirium for β-blockers was 10 days (IQR: 0–121 days). In head-to-head analyses among β-blockers, propranolol was associated with a significantly higher risk of delirium compared to atenolol (aROR: 7.26, 95% CI: 2.57–20.48).

A summary of aRORs with 95% confidence intervals is shown in Tables 2 and 3. Forest plots are presented in the Supplemental Materials (Appendix Figures 9 and 10).

#### Insomnia

We evaluated the risk of insomnia associated with β-blockers (propranolol, atenolol, metoprolol, and carvedilol) compared to lisinopril and losartan. β-blockers showed a significant association with insomnia compared to both lisinopril and losartan.

Among individual β-blockers, carvedilol had the highest association with insomnia compared to lisinopril, followed by propranolol, atenolol, and metoprolol. In comparison with losartan, propranolol showed the highest association with insomnia, followed by atenolol, carvedilol, and metoprolol.

The aROR for insomnia between propranolol and atenolol was 1.06 (95% CI: 0.81–1.30). A summary of aRORs with 95% confidence intervals is shown in Tables 2 and 3. Forest plots are presented in the Supplemental Materials (Appendix Figures 11 and 12).

#### Hallucination

We evaluated the risk of hallucinations associated with β-blockers (propranolol, atenolol, metoprolol, and carvedilol) compared to lisinopril and losartan. β-blockers showed a significant association with hallucinations compared to both lisinopril and losartan.

Among individual β-blockers, propranolol had the highest association with hallucinations compared to lisinopril and losartan, followed by metoprolol. Atenolol and carvedilol showed a moderate to nonsignificant association with both comparators.

In head-to-head analyses among β-blockers, propranolol was associated with a significantly higher risk of hallucinations compared to atenolol (aROR: 2.49, 95% CI: 1.63–3.82).

A summary of aRORs with 95% confidence intervals is shown in Tables 2 and 3. Forest plots are presented in the Supplemental Materials (Appendix Figures 13 and 14).

#### Somnolence

We assessed the risk of somnolence associated with β-blockers (propranolol, atenolol, metoprolol, and carvedilol) compared to lisinopril and losartan. β-blockers were significantly associated with somnolence compared to both lisinopril and losartan.

Among individual β-blockers, carvedilol had the strongest association with somnolence compared to lisinopril, followed by propranolol, metoprolol, and atenolol. In comparison with losartan, propranolol showed the highest association with somnolence, followed by carvedilol, metoprolol, and atenolol.

The aROR for somnolence between propranolol and atenolol was 1.24 (95% CI: 0.94–1.63).

A summary of aRORs with 95% confidence intervals is shown in Tables 2 and 3. Forest plots are presented in the Supplemental Materials (Appendix Figures 15 and 16).

#### Disorientation

We assessed the risk of disorientation associated with β-blockers (propranolol, atenolol, metoprolol, and carvedilol) compared to lisinopril and losartan. β-blockers showed a significant association with disorientation compared to both lisinopril and losartan.

Among individual β-blockers, propranolol had the highest association with disorientation compared to both comparators, followed by metoprolol. Atenolol showed no significant association with lisinopril and losartan.

In head-to-head analyses among β-blockers, propranolol was associated with a significantly higher risk of disorientation compared to atenolol (aROR: 2.12, 95% CI: 1.13–3.96).

A summary of aRORs with 95% confidence intervals is shown in Tables 2 and 3. Forest plots are presented in the Supplemental Materials (Appendix Figures 17 and 18).

#### Confusion

We evaluated the risk of confusion associated with β-blockers (propranolol, atenolol, metoprolol, and carvedilol) compared to lisinopril and losartan. No significant difference in confusion reports was observed between β-blockers and lisinopril, or compared to losartan.

Among individual β-blockers, metoprolol showed a significant association with confusion compared to lisinopril. However, propranolol, atenolol, and carvedilol showed no significant associations with confusion compared to lisinopril. When compared to losartan, carvedilol had a significant association with confusion, while the other β-blockers (propranolol, atenolol, and metoprolol) showed no significant associations.

The aROR for confusion between propranolol and atenolol was 0.99 (95% CI: 0.69–1.44). A summary of aRORs with 95% confidence intervals is shown in Tables 2 and 3. Forest plots are presented in the Supplemental Materials (Appendix Figures 19 and 20).

#### Altered mental status

We evaluated the risk of altered mental status associated with β-blockers (propranolol, atenolol, metoprolol, and carvedilol) compared to lisinopril and losartan. When compared to lisinopril, altered mental status reports for β-blockers were significantly elevated. Propranolol exhibited the highest aROR, followed by metoprolol. Atenolol and carvedilol also showed significant associations.

In comparison to losartan, no significant reports were observed for β-blockers. However, Propranolol and carvedilol showed significant associations, while atenolol and metoprolol did not.

In a head-to-head comparison between propranolol and atenolol, the aROR was 1.02 (95% CI: 0.53–1.98). A summary of aRORs with 95% confidence intervals is shown in Tables 2 and 3. Forest plots are presented in the Supplemental Materials (Appendix Figures 21 and 22).

A post hoc sensitivity analysis was conducted for Nervous System Disorders and Psychiatric Disorders outcomes to evaluate the potential confounding effect of non-cardiovascular indications for beta-blocker use, including migraine, anxiety, and hemangiomas. Adjustment for these additional variables yielded results that were consistent with our primary analysis, indicating that non-cardiovascular uses did not materially alter the observed (Appendix 2).

## Discussion

This study evaluated the neuropsychiatric AEs associated with β-blockers compared to commonly prescribed antihypertensive drugs, lisinopril (an ACE inhibitor) and losartan (an ARB), using data from the FAERS database. Our findings suggest that β-blockers are linked to a significantly higher risk of nervous and psychiatric disorders compared to both lisinopril and losartan. Of the nine types of neuropsychiatric events studied, β-blockers showed higher aRORs for six types, except for confusion, delirium, and altered mental status. In addition, the risk varied across different β-blockers. Propranolol, a lipophilic β-blocker, exhibited the highest aRORs for psychiatric disorders and six types of neuropsychiatric events, including nightmares, delirium, hallucinations, disorientation, altered mental status, and somnolence, compared to both lisinopril and losartan. However, the association remained significant for delirium, hallucinations, and disorientation when comparing propranolol with atenolol, a hydrophilic β-blocker.

Taken together, these findings support prior studies suggesting that lipophilic β-blockers, such as propranolol, can more easily cross the blood–brain barrier, increasing the likelihood of neuropsychiatric adverse effects. In line with our earlier meta-analysis, β-blockers were associated with several neuropsychiatric events, except for dizziness, where an increased risk was observed. Interestingly, in our present study, propranolol was associated with a reduced risk of dizziness compared to losartan, lisinopril, and atenolol, despite expectations that its CNS penetration would increase the risk of dizziness. One possible explanation is that dizziness may result from reduced blood pressure caused by β-blockers, and patients may attribute it to blood pressure fluctuations rather than as an AE. Furthermore, dizziness can be caused by various factors, including sleep disturbances, anxiety, or other health conditions, leading patients to overlook it as a drug AE. In addition, propranolol’s therapeutic use in treating migraines and anxiety could influence patients’ perception of dizziness, as they might experience relief from primary symptoms without recognizing the milder side effects.

Although propranolol was associated with the highest aROR among β-blockers for six types of neuropsychiatric disorders, and the association remained for three of them when compared to atenolol, the lack of statistical power may explain the absence of significant differences in some cases. In addition, it is possible that β-blockers could increase the risk of neuropsychiatric AEs through mechanisms other than their β-blocker properties, such as by altering neurotransmitter levels or other pathways (Cojocariu et al., 2021; McAinsh and Cruickshank, 1990).

The strength of this study lies in its use of real-world data from FAERS, which allowed for a comprehensive analysis of AE patterns across a large and diverse patient population. FAERS, as a valuable pharmacovigilance tool, helps identify common and rare AEs that might not be captured in randomized controlled clinical trials, offering important insights into the safety profile of β-blockers in clinical practice. We applied an active disproportionality analysis to compare β-blockers with lisinopril and losartan, effectively mitigating concerns about false positives. However, there are several limitations to consider. First, FAERS data are subject to underreporting, which could lead to a bias in the findings, especially for milder or less recognized ADEs. Second, notoriety bias could result in overreporting, where certain drugs or events are disproportionately reported due to heightened awareness or publicity. Third, although we adjusted for several known confounders, the self-reported nature of the FAERS database inherently limits our ability to fully account for all potential sources of confounding, particularly patient comorbidities and concurrent medications. To minimize confounding by indication, we adjusted for key demographic and clinical variables, including age, sex, weight, and common cardiovascular indications for beta-blockers, lisinopril, and losartan (i.e., hypertension, heart failure, and atrial fibrillation). However, we acknowledge that residual confounding remains possible, as indications for use are not systematically or uniformly reported in FAERS.

To address this limitation further, we conducted a post hoc sensitivity analysis in which we additionally adjusted for non-cardiovascular indications for beta-blocker use—such as migraine, anxiety, hemangiomas—alongside cardiovascular indications and patient demographics. These non-cardiovascular indications were extracted from the “indication for use” field in FAERS reports when available. Importantly, the results of this expanded analysis remained consistent with our primary findings (see Appendix 2) in Supplemental files.

While the overall median age of patients reporting β-blocker-related AEs was comparable to those reporting events for lisinopril or losartan, regardless of the indication of use, the propranolol subgroup displayed a younger age distribution. This pattern is consistent with its off-label non-cardiac uses, including treatment for infantile hemangiomas, performance anxiety, and migraine. Although this variation in age may introduce potential confounding, age was included as a covariate in all adjusted models, which likely reduced—but may not have fully eliminated—this concern. Importantly, the consistency of results in sensitivity analyses adjusting for both cardiac and non-cardiac indications further supports the robustness of our findings.

Fourth, while the FAERS database allows us to capture ADEs and their temporal relationships with specific medications, it does not provide detailed information on pre-existing psychiatric or neurological conditions in the patient population. This limitation arises from the nature of pharmacovigilance databases, which are designed primarily to detect a signal ADE, rather than to offer comprehensive clinical data. As such, we were unable to account for existing psychiatric or neurological diagnoses as confounders or covariates in our analysis, which may have introduced residual confounding and influenced the associations observed. Lastly, we acknowledge the potential for spurious associations due to multiple comparisons in our analysis. However, as this study was exploratory and aimed at hypothesis generation, we intentionally did not adjust for Type I errors. Such adjustments could obscure potentially meaningful signals that merit further investigation. The findings from this study will be rigorously tested in a subsequent population-based study to confirm their validity. In summary, our findings suggest that β-blockers may be associated with a higher risk of neuropsychiatric AEs compared to lisinopril and losartan. The risk varies depending on the specific β-blocker and the type of neuropsychiatric event. Propranolol, a lipophilic β-blocker, demonstrated the highest aRORs and was most strongly linked to a range of psychiatric AEs. However, these findings should be viewed as generating hypotheses that warrant confirmation or refutation in future population-based studies.

## Supplemental Material

sj-docx-1-jop-10.1177_02698811251349190 – Supplemental material for β-blockers and risk of neuropsychiatric adverse events: An active-comparator restricted disproportionality on the FAERS Supplemental material, sj-docx-1-jop-10.1177_02698811251349190 for β-blockers and risk of neuropsychiatric adverse events: An active-comparator restricted disproportionality on the FAERS by Lujain Ez Eddin, Mohammad Ali Omrani, Niaz Chalabianloo and Flory T Muanda in Journal of Psychopharmacology
