# Supplementary material for: β-blockers and risk of neuropsychiatric adverse events: An active-comparator restricted disproportionality on the FAERS
Source: J Psychopharmacol. 2025 Jul 24;39(11):1299–306. doi: 10.1177/02698811251349190 (PMC12618733; doi:10.1177/02698811251349190)
Supplement: sj-docx-1-jop-10.1177_02698811251349190 – Supplemental material for β-blockers and risk of neuropsychiatric adverse events: An active-comparator restricted disproportionality on the FAERS [file sj-docx-1-jop-10.1177_02698811251349190.docx]

**Appendix 1**

1. **Nervous System Disorder**


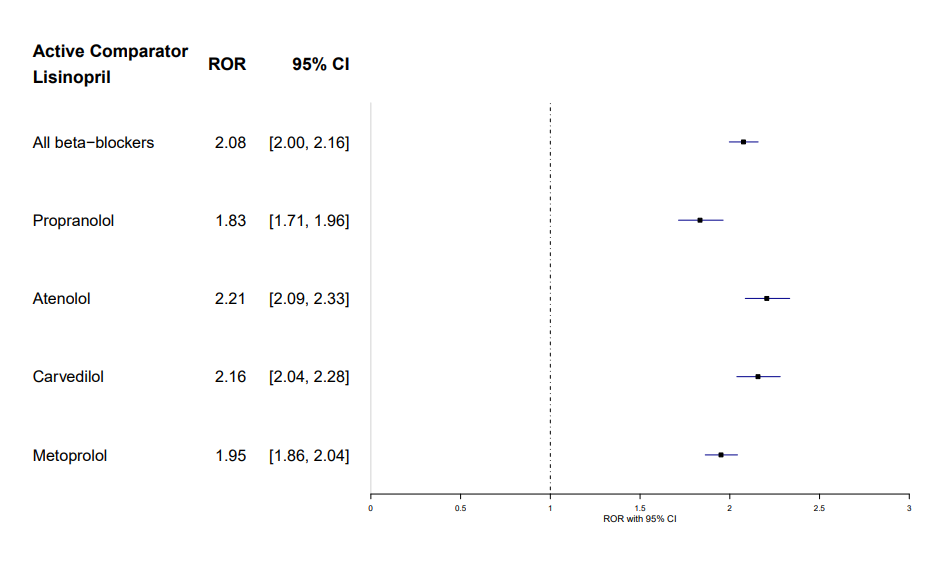


Figure 1: Forest Plot Reporting Odds Ratios (RORs) and 95% Confidence Intervals for Nervous System Disorders Associated with All β-Blockers and Individual β-Blocker Agents Compared to Lisinopril.


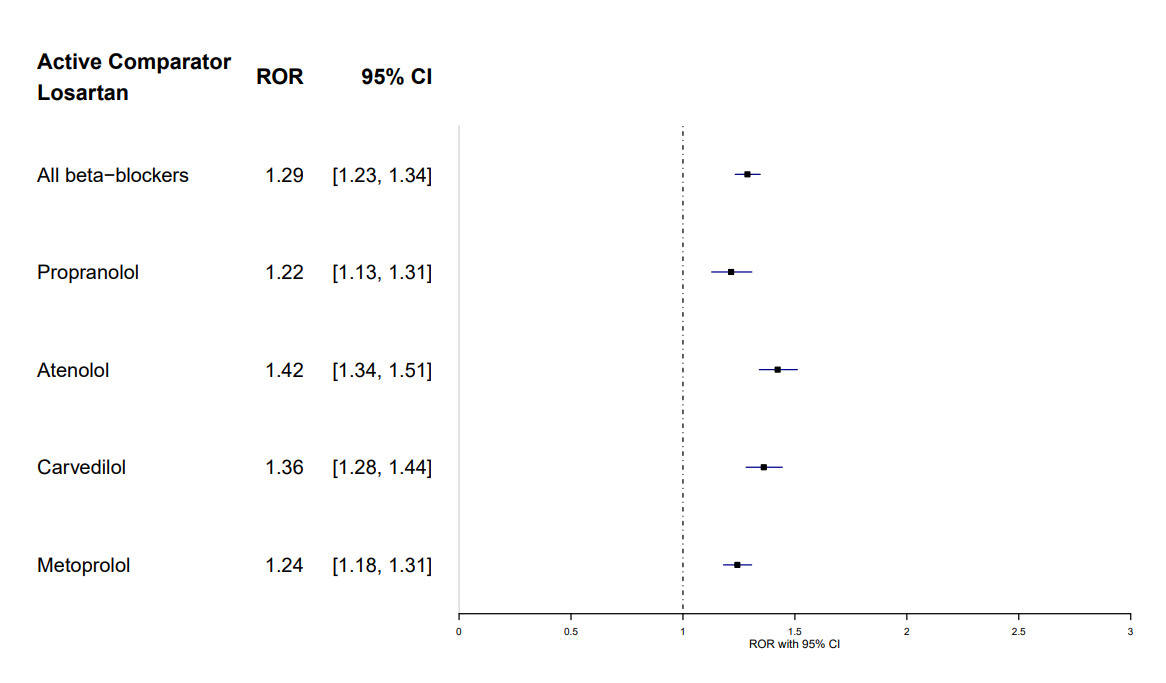


Figure 2: Forest Plot Reporting Odds Ratios (RORs) and 95% Confidence Intervals for Nervous System Disorders Associated with All β-Blockers and Individual β-Blocker Agents Compared to Losartan.

1. **Psychiatric Disorder**

**
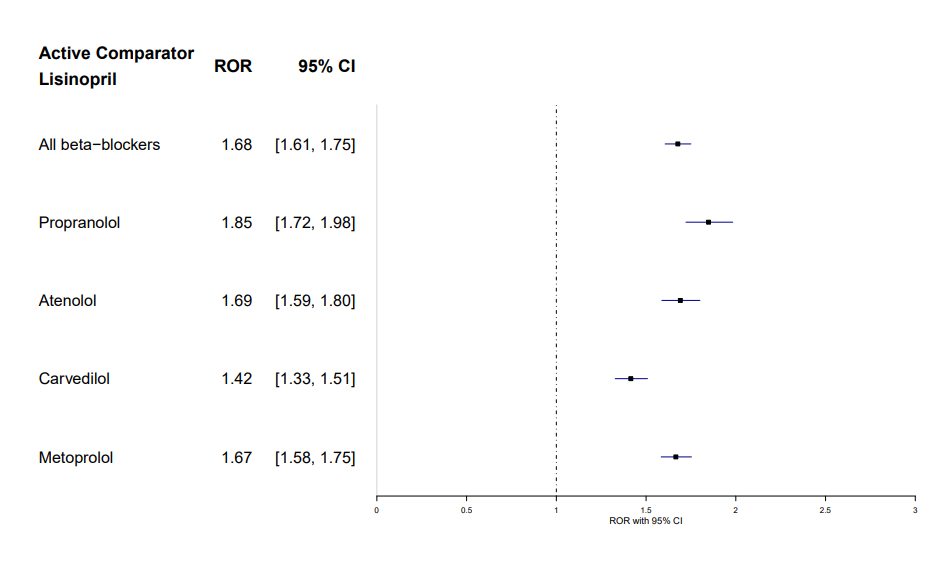
**

Figure 3: Forest Plot Reporting Odds Ratios (RORs) and 95% Confidence Intervals for Psychiatric Disorders Associated with All β-Blockers and Individual β-Blocker Agents Compared to Lisinopril.

**
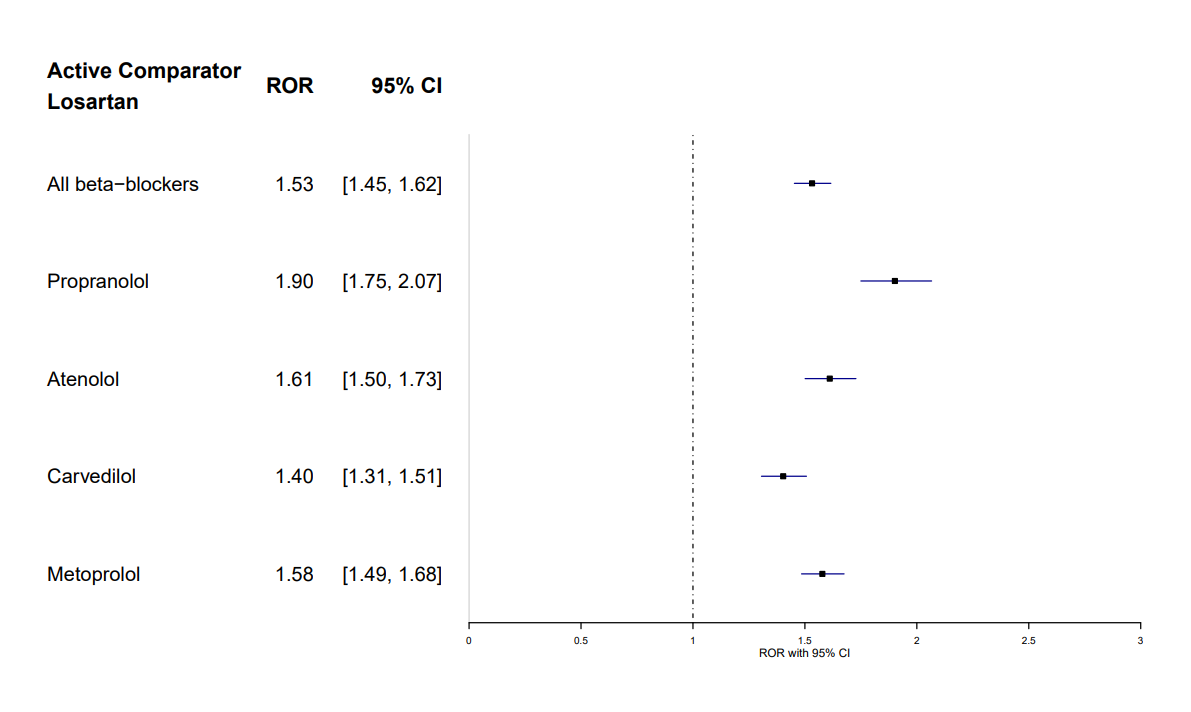
**

Figure 4: Forest Plot Reporting Odds Ratios (RORs) and 95% Confidence Intervals for Psychiatric Disorders Associated with All β-Blockers and Individual β-Blocker Agents Compared to Losartan.

1. **Dizziness**

**
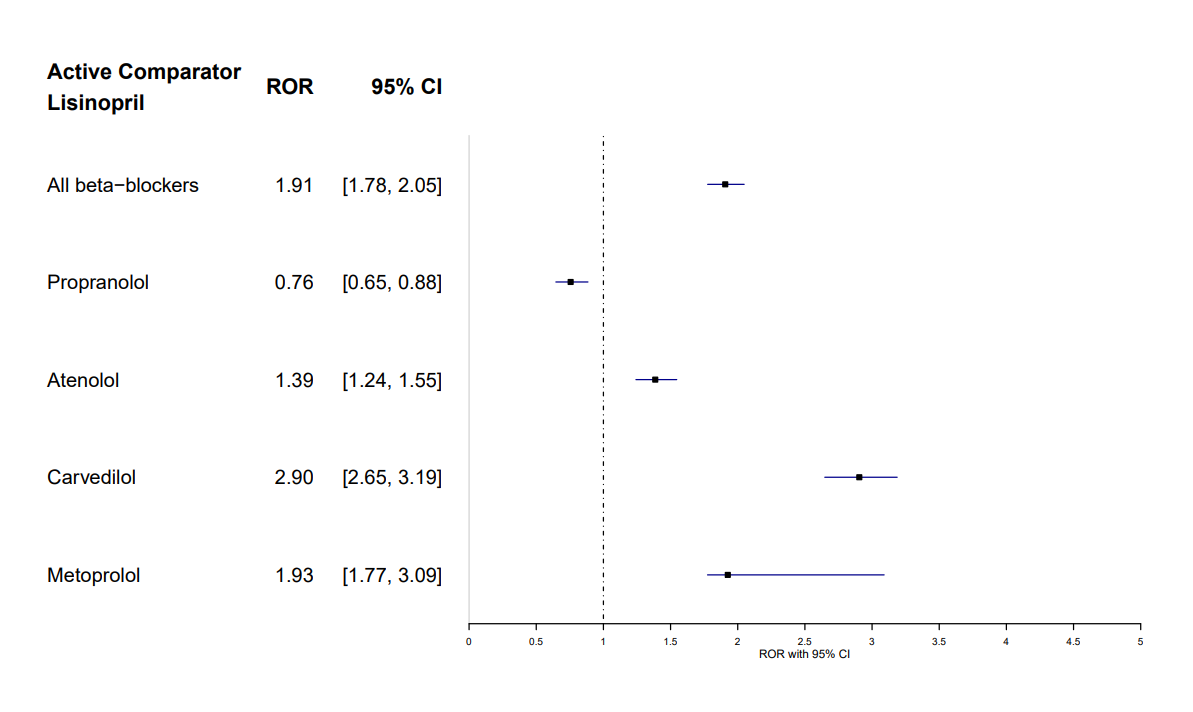
**

Figure 5: Forest Plot Reporting Odds Ratios (RORs) and 95% Confidence Intervals for Dizziness Associated with All β-Blockers and Individual β-Blocker Agents Compared to Lisinopril.

**
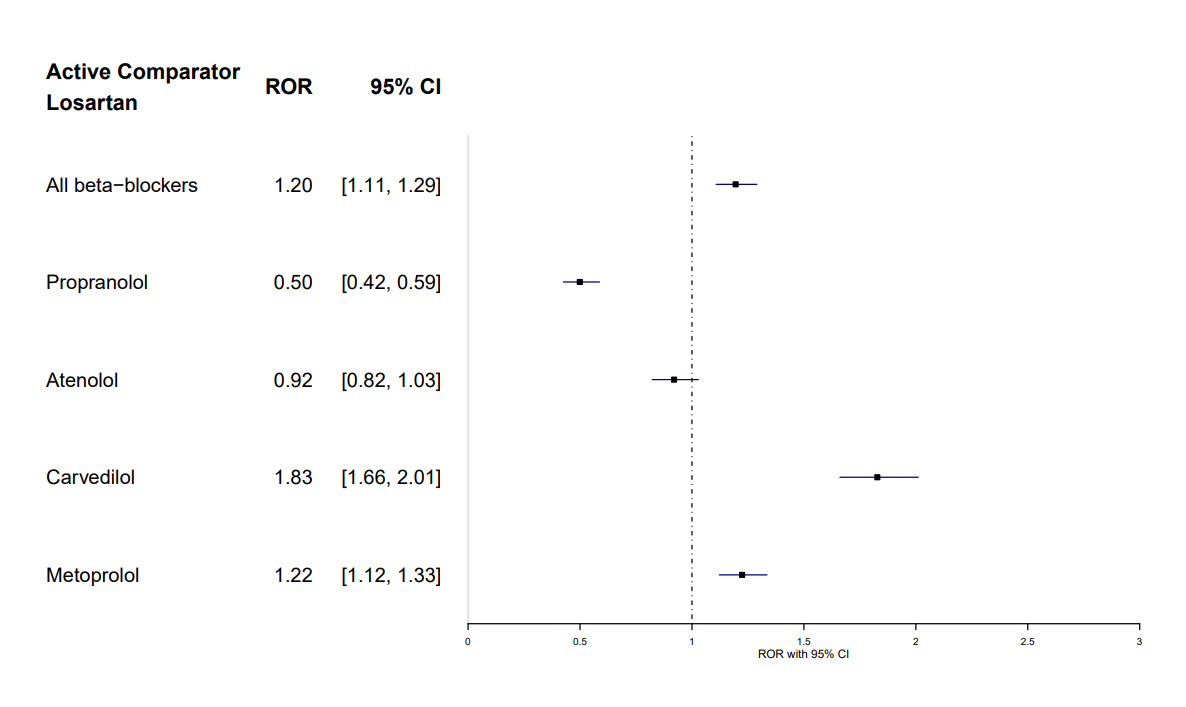
**

Figure 6: Forest Plot Reporting Odds Ratios (RORs) and 95% Confidence Intervals for Dizziness Associated with All β-Blockers and Individual β-Blocker Agents Compared to Losartan.

1. **Nightmares**

**
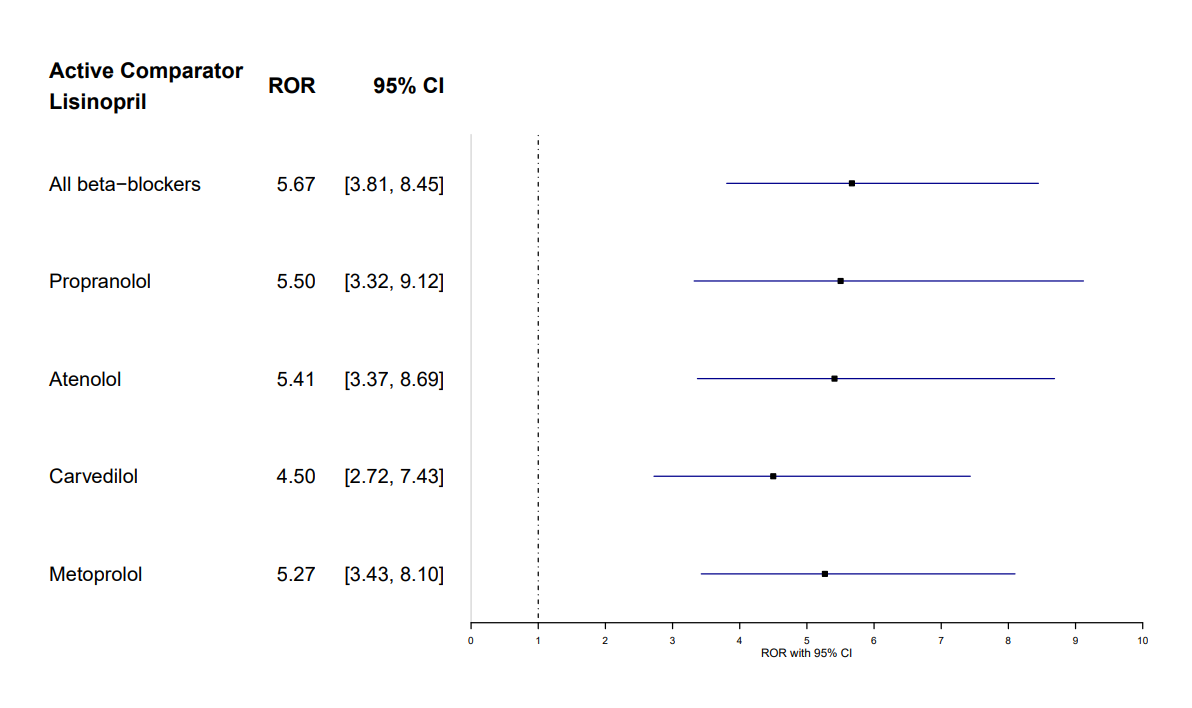
**

Figure 7: Forest Plot Reporting Odds Ratios (RORs) and 95% Confidence Intervals for Nightmares Associated with All β-Blockers and Individual β-Blocker Agents Compared to Lisinopril.

**
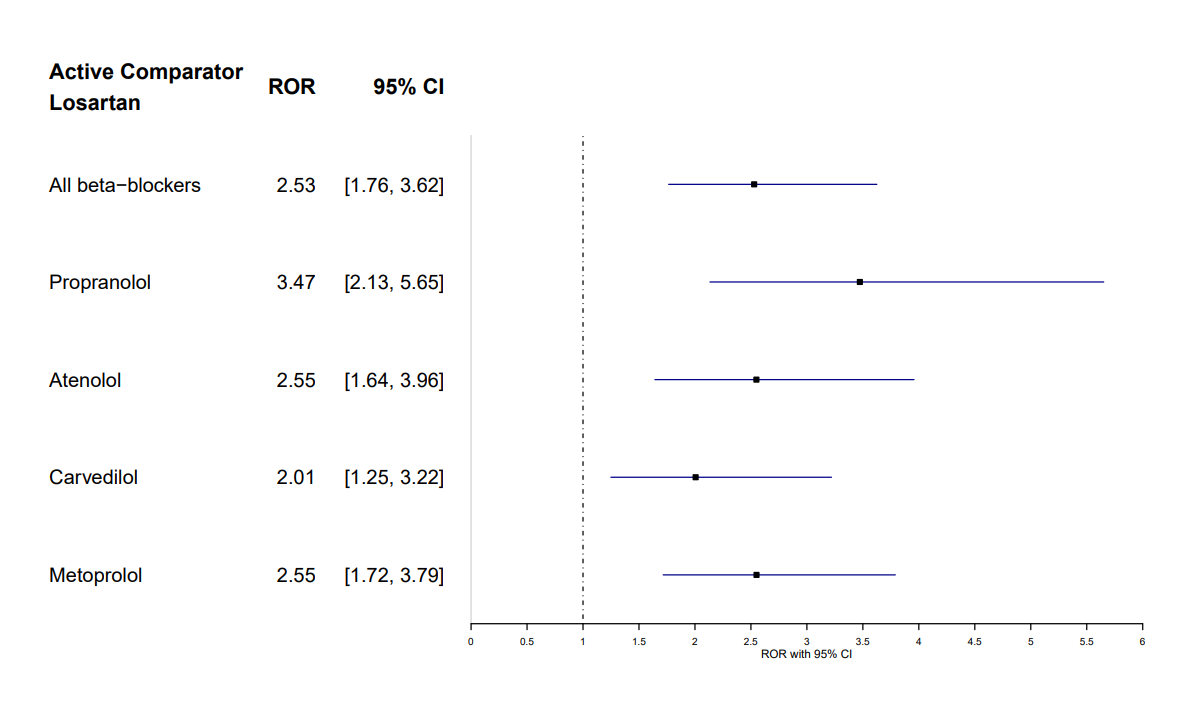
**

Figure 8: Forest Plot Reporting Odds Ratios (RORs) and 95% Confidence Intervals for Nightmares Associated with All β-Blockers and Individual β-Blocker Agents Compared to Losartan.

1. **Delirium**

**
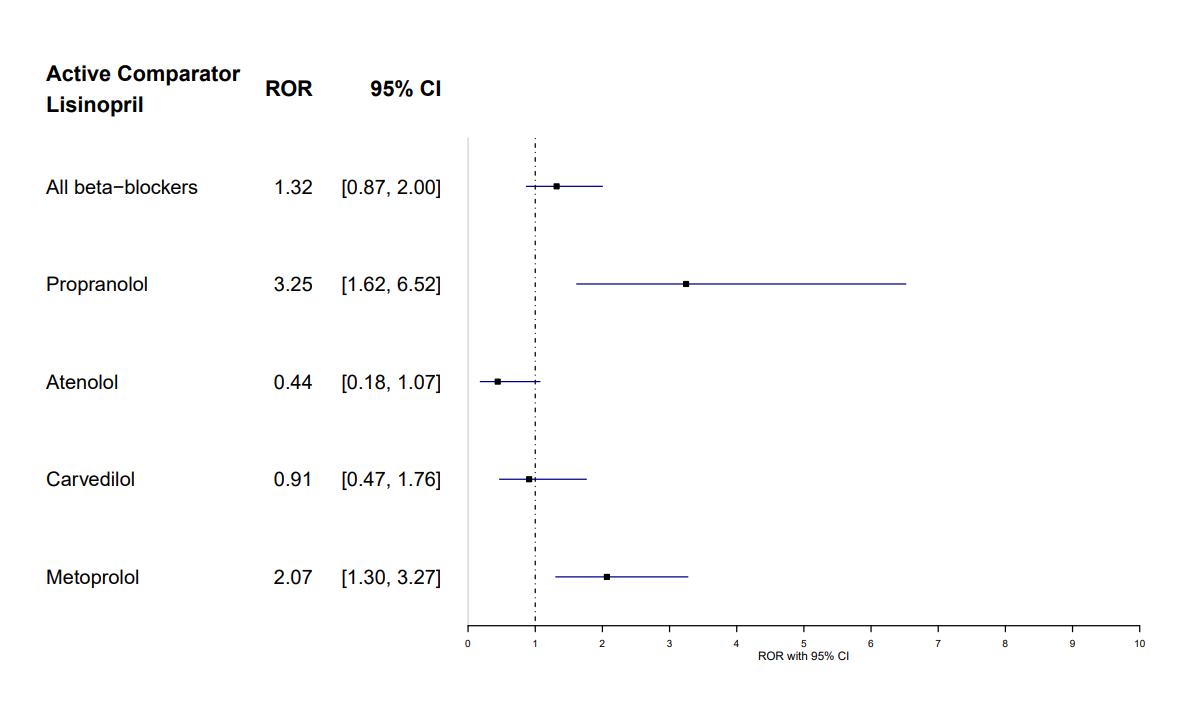
**

Figure 9: Forest Plot Reporting Odds Ratios (RORs) and 95% Confidence Intervals for Delirium Associated with All β-Blockers and Individual β-Blocker Agents Compared to Lisinopril.

**
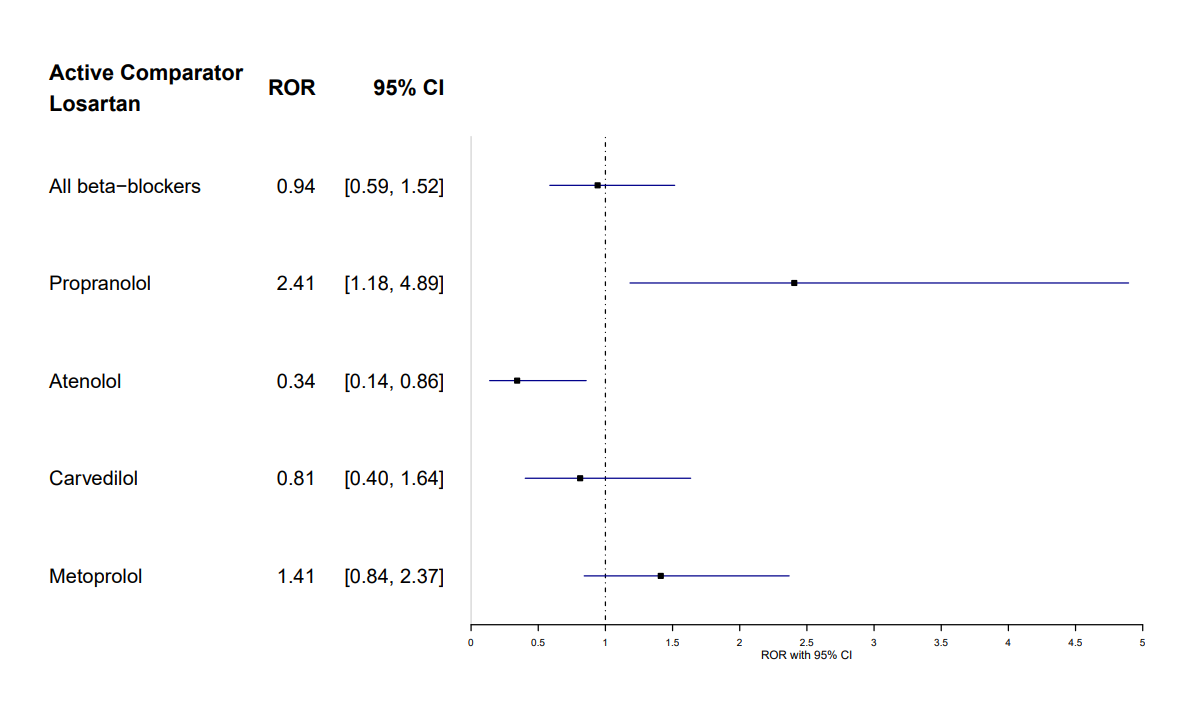
**

Figure 10: Forest Plot Reporting Odds Ratios (RORs) and 95% Confidence Intervals for Delirium Associated with All β-Blockers and Individual β-Blocker Agents Compared to Losartan.

1. **Insomnia**

**
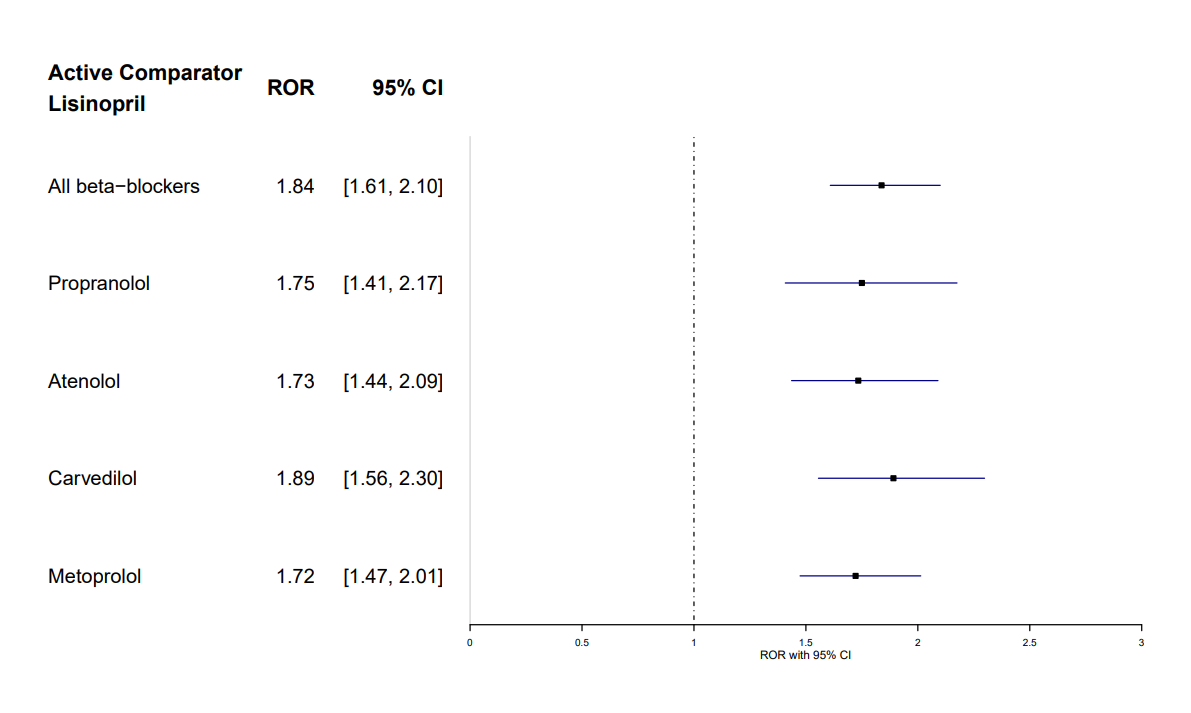
**

Figure 11: Forest Plot Reporting Odds Ratios (RORs) and 95% Confidence Intervals for Insomnia Associated with All β-Blockers and Individual β-Blocker Agents Compared to Lisinopril.

**
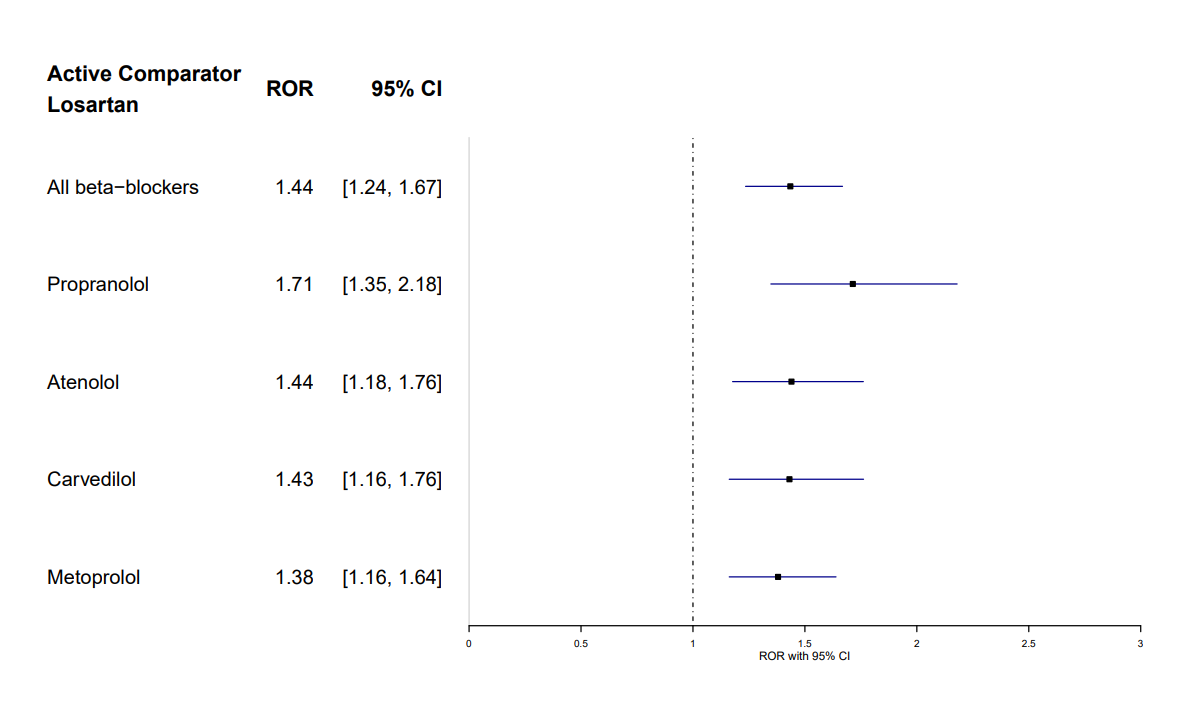
**

Figure 12: Forest Plot Reporting Odds Ratios (RORs) and 95% Confidence Intervals for Insomnia Associated with All β-Blockers and Individual β-Blocker Agents Compared to Losartan.

1. **Hallucinations**

**
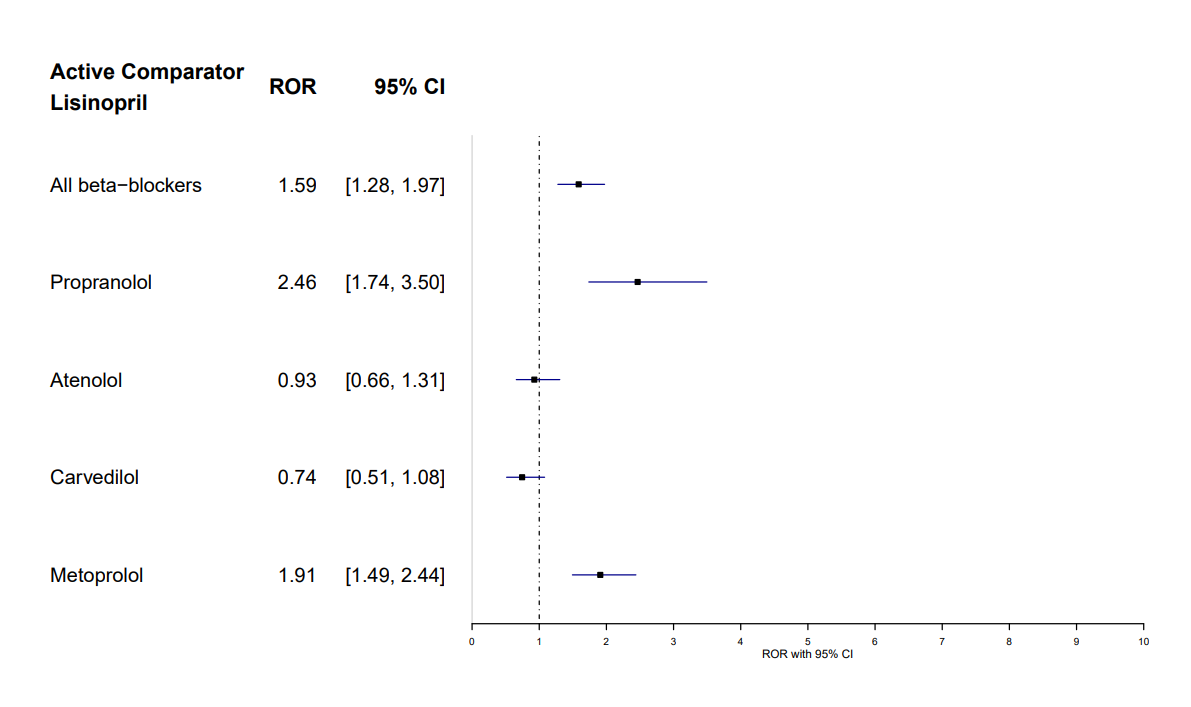
**

Figure 13: Forest Plot Reporting Odds Ratios (RORs) and 95% Confidence Intervals for Hallucination Associated with All β-Blockers and Individual β-Blocker Agents Compared to Lisinopril.

**
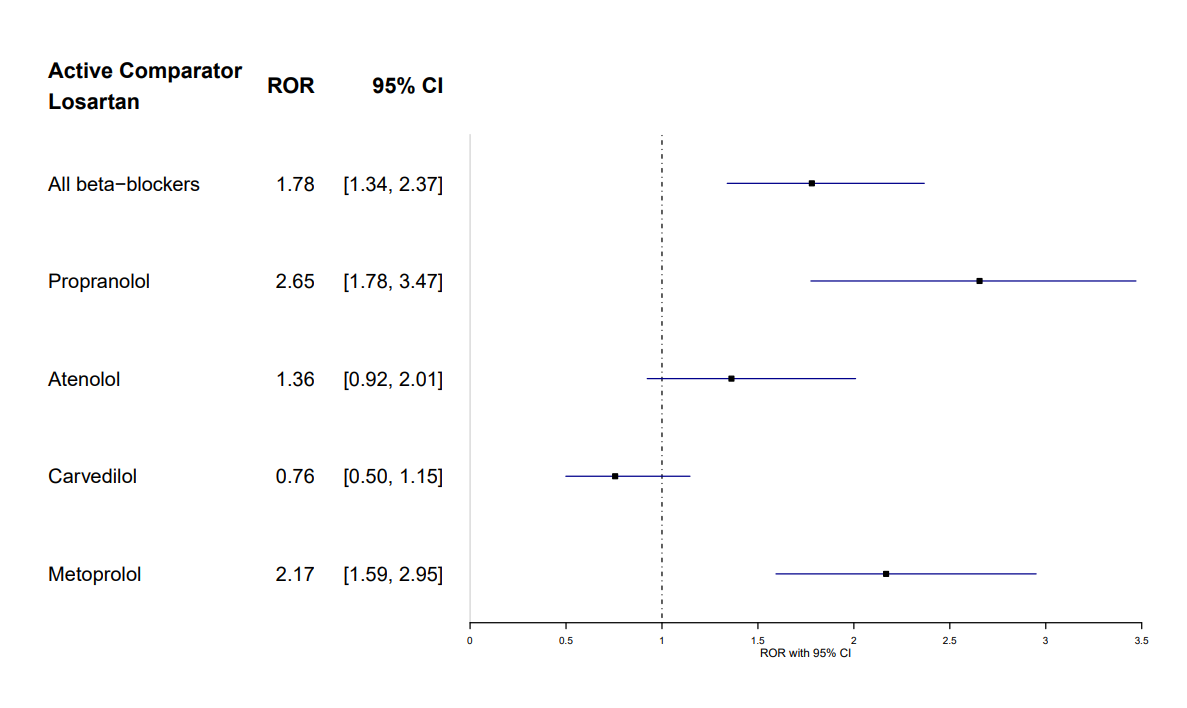
**

Figure 14: Forest Plot Reporting Odds Ratios (RORs) and 95% Confidence Intervals for Hallucination Associated with All β-Blockers and Individual β-Blocker Agents Compared to Losartan.

1. **Somnolence**

**
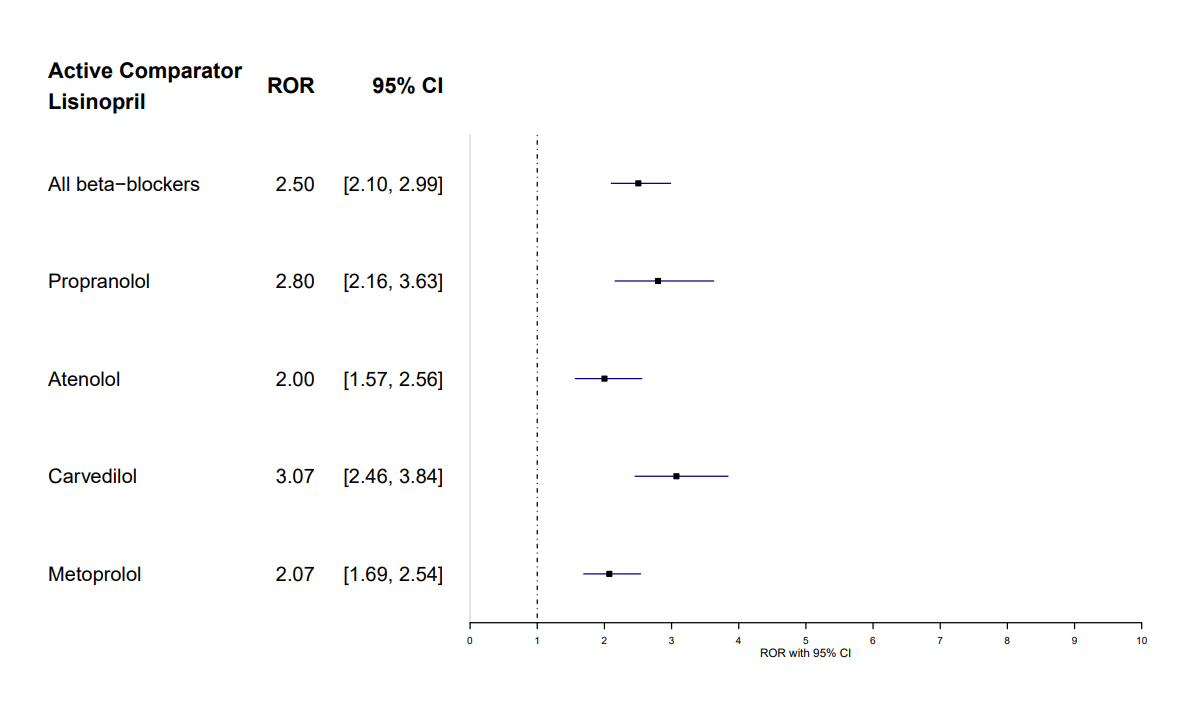
**

Figure 15: Forest Plot Reporting Odds Ratios (RORs) and 95% Confidence Intervals for Somnolence Associated with All β-Blockers and Individual β-Blocker Agents Compared to Lisinopril.

**
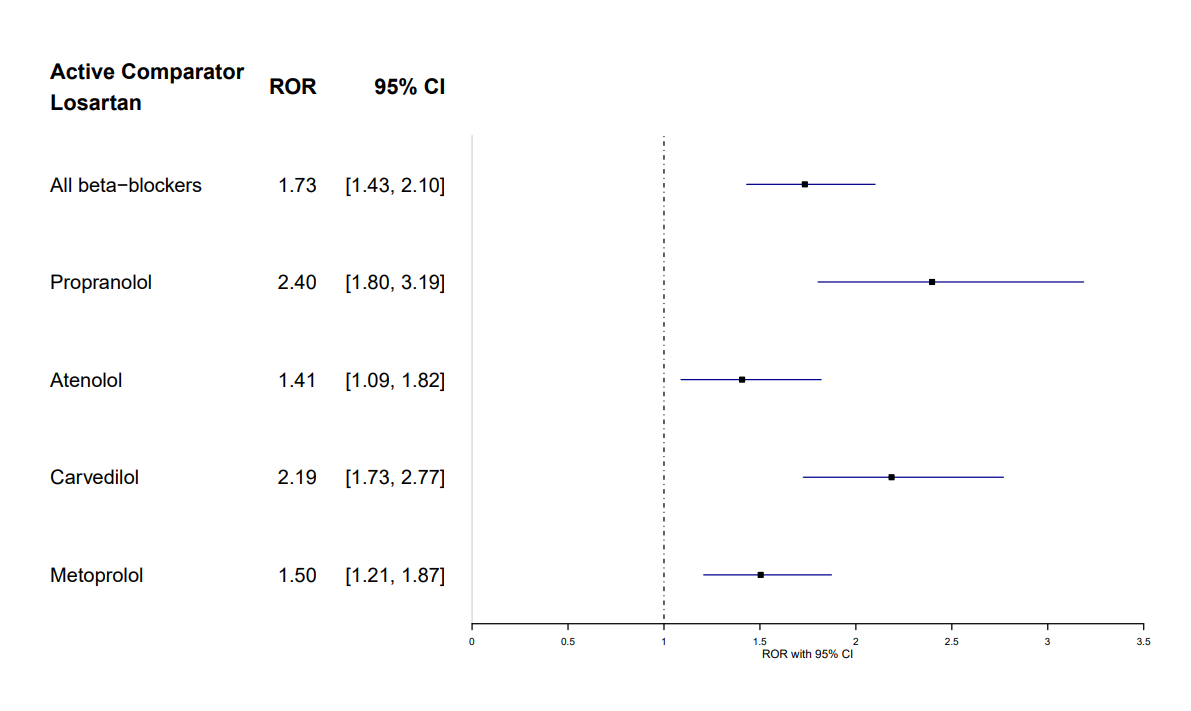
**

Figure 16: Forest Plot Reporting Odds Ratios (RORs) and 95% Confidence Intervals for Somnolence Associated with All β-Blockers and Individual β-Blocker Agents Compared to Losartan.

1. **Disorientation**

**
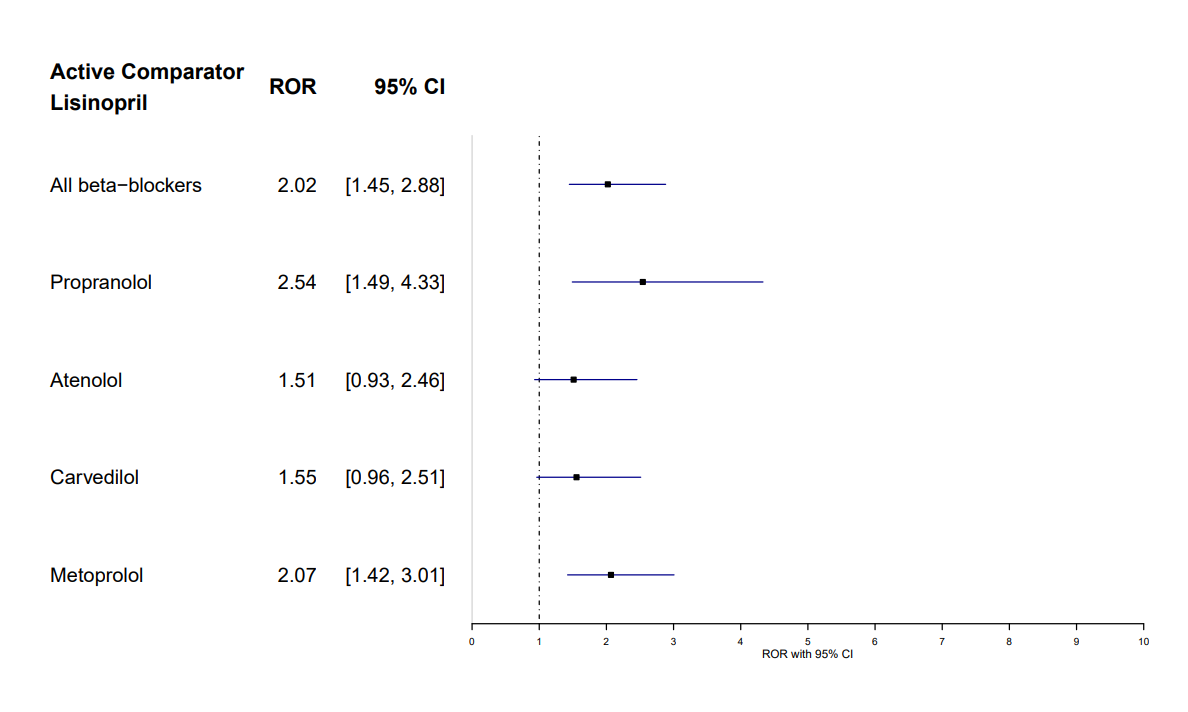
**

Figure 17: Forest Plot Reporting Odds Ratios (RORs) and 95% Confidence Intervals for Disorientation Associated with All β-Blockers and Individual β-Blocker Agents Compared to Lisinopril.

**
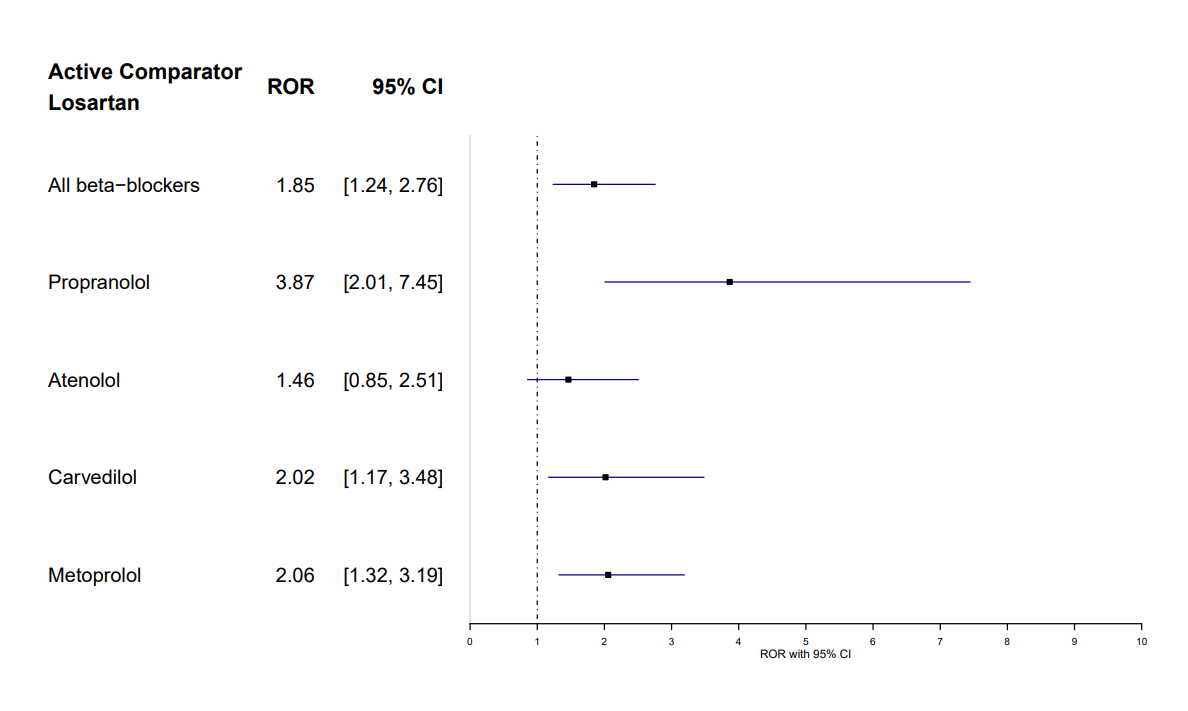
**

Figure 18: Forest Plot Reporting Odds Ratios (RORs) and 95% Confidence Intervals for Disorientation Associated with All β-Blockers and Individual β-Blocker Agents Compared to Losartan.

1. **Confusion**

**
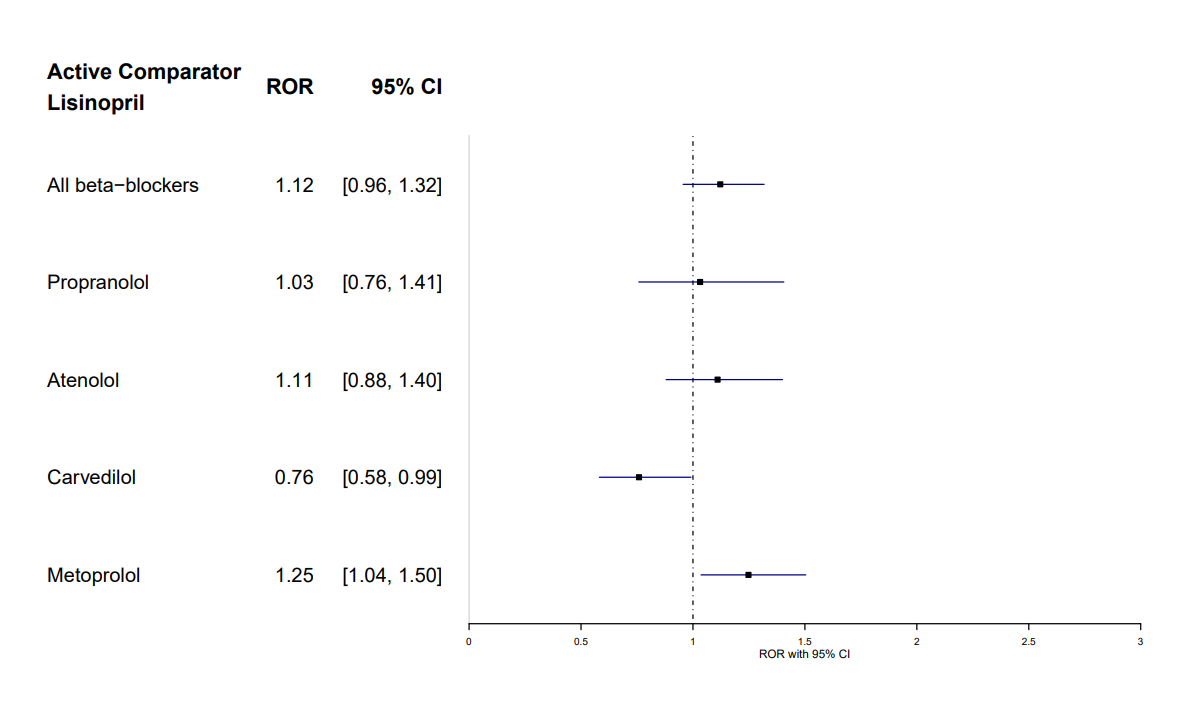
**

Figure 19: Forest Plot Reporting Odds Ratios (RORs) and 95% Confidence Intervals for Confusion Associated with All β-Blockers and Individual β-Blocker Agents Compared to Lisinopril.

**
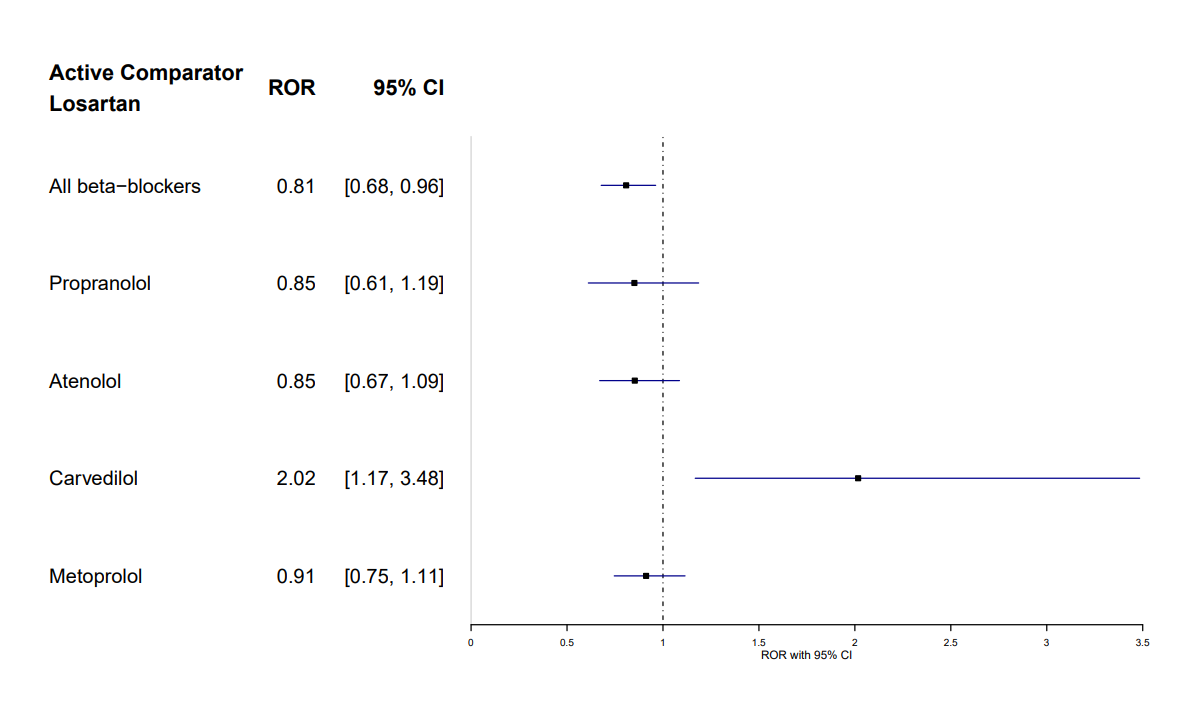
**

Figure 20: Forest Plot Reporting Odds Ratios (RORs) and 95% Confidence Intervals for Confusion Associated with All β-Blockers and Individual β-Blocker Agents Compared to Losartan.

1. **Altered Mental Status**

**
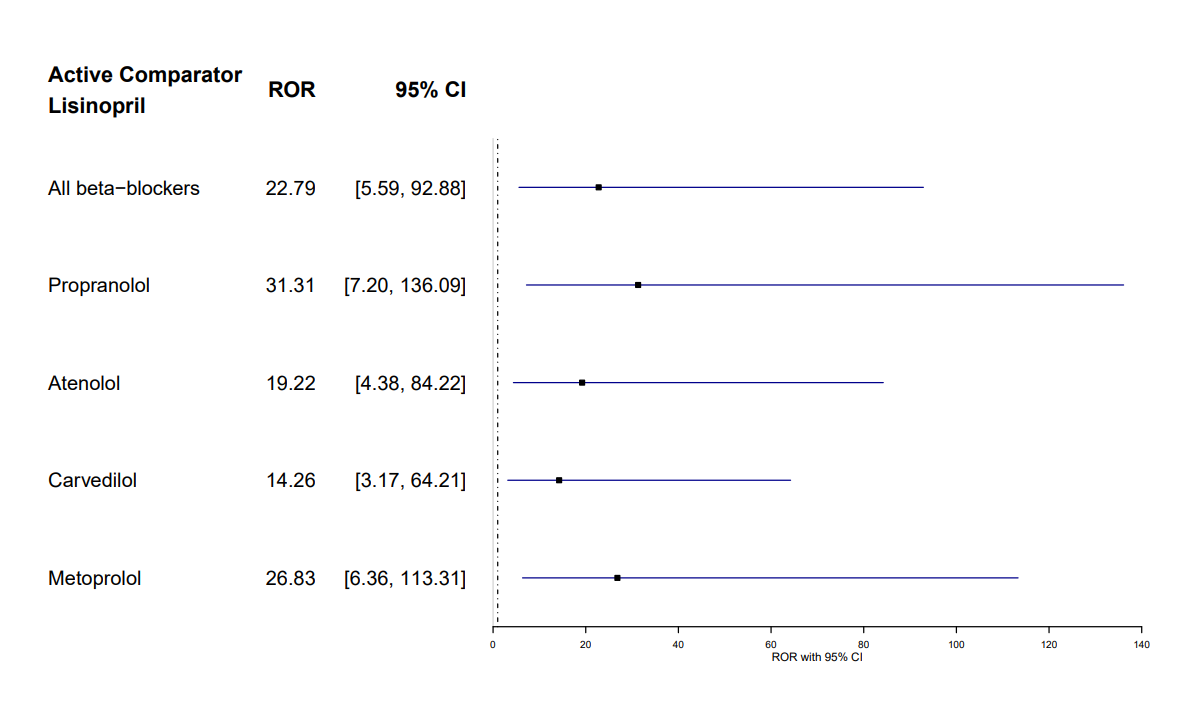
**

Figure 21: Forest Plot Reporting Odds Ratios (RORs) and 95% Confidence Intervals for Altered Mental Status Associated with All β-Blockers and Individual β-Blocker Agents Compared to Lisinopril.

**
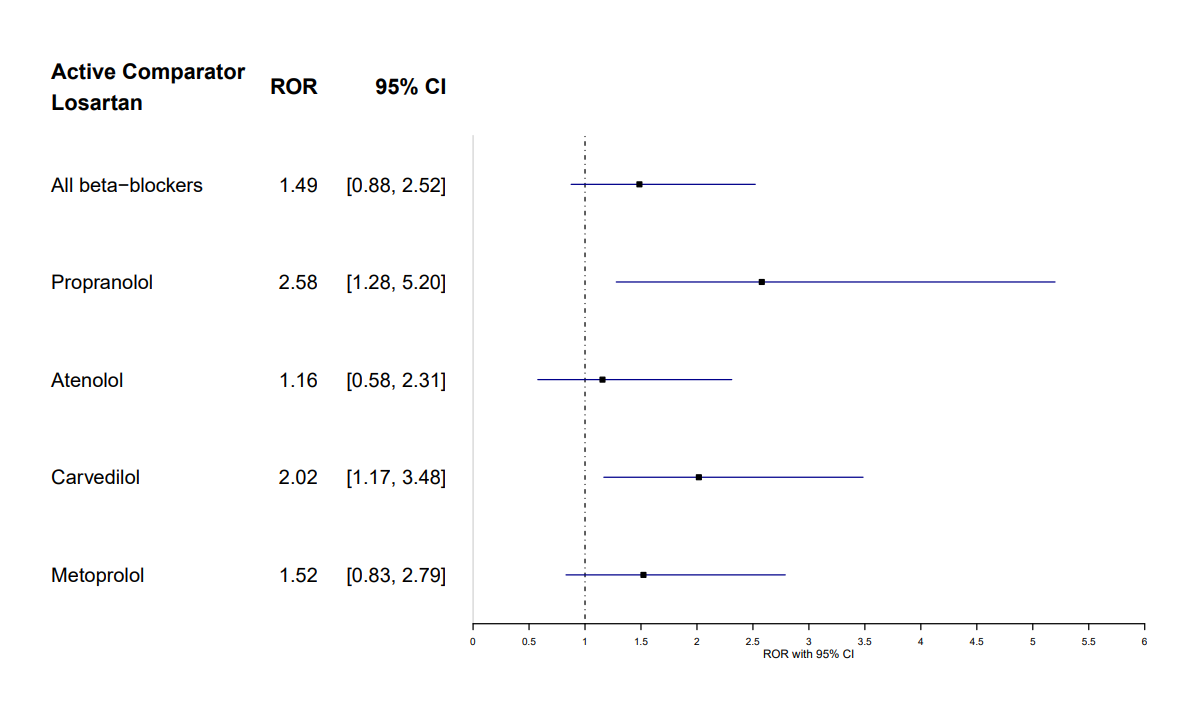
**

Figure 22: Forest Plot Reporting Odds Ratios (RORs) and 95% Confidence Intervals for Altered Mental Status Associated with All β-Blockers and Individual β-Blocker Agents Compared to Losartan.

**Appendix 2**

Table 1 Comparison of the Risk of Neuropsychiatric Adverse Events Between β-blockers and Lisinopril after Adjusting for Migraine, Anxiety, Hemangiomas, Sex, Age, Weight, Hypertension, Heart Failure, and Atrial Fibrillation.

| Adverse event | β-blockers  aROR [95% CI] |
| --- | --- |
| **Nervous System Disorders** | 2.09 [2.01, 2.17] |
| **Psychiatric Disorders** | 1.67 [1.60, 1.74] |

Table 2 Comparison of the Risk of Neuropsychiatric Adverse Events Between β-blockers and Losartan after Adjusting for Migraine, Anxiety, Hemangiomas, Sex, Age, Weight, Hypertension, Heart Failure, and Atrial Fibrillation.

| Adverse event | β-blockers  aROR [95% CI] |
| --- | --- |
| **Nervous System Disorders** | 1.29 [1.24, 1.35] |
| **Psychiatric Disorders** | 1.53 [1.45, 1.62] |

**Appendix 3**

**The READUS-PV checklist for abstracts**

| **Section and topic** | **Item #** | **Checklist item** | **Location where item is reported** |
| --- | --- | --- | --- |
| Background | *1a* | *State the aim/rationale for performing the study.* | *Page 2 (lines 35-38)* |
|  | *1b* | *Specify the adverse event(s) and/or the drug(s) under study, when applicable.* | *Page 2 (lines 33-34)* |
|  | *1c* | *Specify the specific population or setting, when applicable.* | *Not applicable* |
| Methods | *2a* | *Identify the study as a “disproportionality analysis” and specify the type of data used.* | *Page 2 (lines 39-40)* |
|  | *2b* | *Specify the name of the database(s) used and the type of access.* | *Page 2 (lines 40-41)* |
|  | *2c* | *Specify the timeframe and geographical region, when applicable.* | *Page 2 (line 40)* |
|  | *2d* | *Specify the disproportionality measure(s) used and their statistical significance threshold(s).* | *Page (lines 46-48)* |
|  | *2e* | *Specify if a case-by-case analysis is performed.* | *Not applicable* |
| Results | *3* | *Report main findings including their precision (e.g., 95% confidence intervals), together with a short summary of the case-by-case analysis.* | *Page 2-3 (lines 49-57)* |
| Conclusion | *4a* | *Clearly report key conclusions.* | *Page 3 (lines 58-61)* |
|  | *4b* | *Acknowledge that the disproportionality analysis is a hypothesis generating or refinement approach.* | *Page 3 (lines 63-64)* |
|  | *4c* | *State the implications and clinical relevance of the findings.* | *Page 3 (lines 61-62)* |

**Appendix 2**

**The READUS-PV checklist**

| **Section and topic** | **Item #** | **Checklist item** | **Location where item is reported** |
| --- | --- | --- | --- |
| **Title** |  |  |  |
|  | *1a* | *If disproportionality analyses are a prominent component of the published study, the study should be identified as a “disproportionality analysis”. The type of data and name of the database(s) should be specified.* | *Page 1 (lines 1-2)* |
|  | *1b* | *Report the name of adverse event(s) and/or drug(s) under study, when applicable.* | *Page 1 (line 1-2)* |
| **Introduction** |  |  |  |
| Background | *2a* | *Describe the drug(s) and its utilization, the nature of the adverse event(s) under study and its frequency, and the existing knowledge on the drug-event combination.* | *Page 4 (lines 76-92)* |
|  | *2b* | *Specify the rationale for performing the analysis, e.g., as part of routine pharmacovigilance, to investigate an overall safety profile, or to assess a pre-specified hypothesis.* | *Page 5 (lines 103-110)* |
|  | *2c* | *Explain why ICSR databases and disproportionality analysis are suitable to fill the knowledge gap.* | *Page 5 (lines 112-119)* |
| Objectives | *3* | *State specific objectives, identifying the adverse event(s), the drug(s), and the reference group, including any pre-specified hypothesis, if applicable.* | *Page 5 (lines 105-118)* |
| **Methods** |  |  |  |
| Study design | *4a* | *Identify the study (i.e., “disproportionality analysis”) and the type of data used (e.g., “individual case safety reports”).* | *Page 6 (lines 121-122)* |
|  | *4b* | *Provide an outline of the entire study design, including primary and sensitivity analyses performed, and other designs such as case-by-case analysis or literature review.* | *Page 6 (lines 126-132)* |
| Data description, access, and pre-processing | *5a* | *Specify the name of the database(s), the database(s) custodian, and the coverage. Specify the type/number of drugs included within the database and the thesaurus, taxonomies, or ontologies used for coding drugs and events.* | *Page 7 (lines 142-150)* |
|  | *5b* | *Specify the extraction dates and describe and justify all choices used for data pre-processing, including any data transformation or exclusion, if appropriate.* | *Page 7 (lines 143-144) and (151-157)* |
| Variables definition | *6a* | *Describe the study population, including any restriction.* | *Page 8 (lines 175-176)* |
|  | *6b* | *Describe the nature and the meaning of key variables assessed in the work.* | *Page 8 (lines184- 186)* |
|  | *6c* | *Specify and justify any grouping of drugs or events. For drugs, specify and justify whether active ingredients/trade names/salts were considered and/or the selected role.* | *Page 9 (lines191-1193)* |
|  | *6d* | *Describe any additional data source used, the type of data, and how they interact with ICSRs.* | *none* |
| Statistical methods | *7a* | *Present any descriptive analysis performed, specifying variables investigated, statistical tests, and significance thresholds.* | *Page 9 (lines 203-207))* |
|  | *7b* | *Describe the measure(s) selected for the disproportionality analysis including any threshold used to identify signals of disproportionate reporting. Explain the reason for this choice if applicable.* | *Page 9 (lines 206-207)* |
|  | *7c* | *Clearly describe any sensitivity analysis and any tool to control confounding, including any restriction, subgroup, stratification, adjustment, or interaction.* | *Page 10 (lines 212- 217)* |
|  | *7d* | *Specify the variables and methods used for the case-by-case analysis, including any algorithm or criteria used to assess causality, if performed.* | *none* |
|  | *7e* | *Specify any statistical methods used for other data sources.* | *none* |
| **Results** |  |  |  |
| Participants | *8a* | *Specify the number of individual case safety reports included at each stage, including reasons for exclusion.* | *Page 10 (lines 221-225)* |
|  | *8b* | *Provide key demographic and clinical characteristics of cases, if possible comparing cases with any appropriate reference group.* | *Page 10 (lines 225-228)* |
| Disproportionality analysis | *9* | *Present all results including confidence intervals. Present also results of sensitivity analyses, if performed.* | *Pages 11-18 (lines 231-381)* |
| Case-by-case analysis | *10* | *Present the case-by-case analysis of key variables. Present the causality assessment, if applicable.* | *Not applicable* |
| **Discussion** |  |  |  |
| Key results | *11* | *Discuss key results with reference to study objectives and contextualize them within the current literature and other consulted sources. Clearly discriminate between expected reactions and emerging safety signals.* | *Page 18 (lines 383- 395)* |
| External validity | *12a* | *Discuss the external validity of the results to the general population.* | *Page 21 (lines 447-448)* |
|  | *12b* | *Discuss the potential relevance of results in clinical practice* | *Page 18-19 (lines 396-402)* |
|  | *12c* | *Propose further study designs if applicable* | *Page 20 (lines 442-443)* |
| Limitations | *13* | *Present general limitations, making clear that disproportionality analysis alone cannot prove causation or measure incidence, and specific limitations, including confounding and reporting bias and efforts to mitigate them.* | *Page 20-21 (lines 421-443)* |
| **Declarations** |  |  |  |
|  | *14a* | *Provide the source of funding/sponsorship and the role of the funders/sponsors for the present study and for any original study on which the present article is based.* | *None* |
|  | *14b* | *Clearly identify potential commercial and intellectual conflicts of interest (e.g., link to any drug/event investigated, whether financial, legal action, or software used).* | *None* |
|  | *14c* | *Declare any institutional approval needed or granted in the investigation.* | *None* |
|  | *14d* | *Include a statement on data availability, code availability (including the version of the statistical software used), and protocol registration.* | *Page 21 (lines 449-460)* |
